# Role of *Arabidopsis* Splicing factor SF1 in Temperature-Responsive Alternative Splicing of *FLM* pre-mRNA

**DOI:** 10.3389/fpls.2020.596354

**Published:** 2020-12-01

**Authors:** Keh Chien Lee, Kyung Sook Chung, Hee Tae Lee, Jae-Hyeok Park, Jeong Hwan Lee, Jeong-Kook Kim

**Affiliations:** ^1^Division of Life Sciences, Korea University, Seoul, South Korea; ^2^Division of Life Sciences, Jeonbuk National University, Jeonju, South Korea

**Keywords:** alternative splicing, ambient temperature, *Arabidopsis thaliana*, *AtSF1*, *FLM*, *FLM-*β, *FLM-δ*, temperature-responsive flowering

## Abstract

Small changes in temperature affect plant ecological and physiological factors that impact agricultural production. Hence, understanding how temperature affects flowering is crucial for decreasing the effects of climate change on crop yields. Recent reports have shown that *FLM-*β, the major spliced isoform of *FLOWERING LOCUS M* (*FLM*)—a flowering time gene, contributes to temperature-responsive flowering in *Arabidopsis thaliana*. However, the molecular mechanism linking pre-mRNA processing and temperature-responsive flowering is not well understood. Genetic and molecular analyses identified the role of an *Arabidopsis* splicing factor *SF1* homolog, *AtSF1*, in regulating temperature-responsive flowering. The loss-of-function *AtSF1* mutant shows temperature insensitivity at different temperatures and very low levels of *FLM-*β transcript, but a significantly increased transcript level of the alternative splicing (AS) isoform, *FLM-δ*. An RNA immunoprecipitation (RIP) assay revealed that AtSF1 is responsible for ambient temperature-dependent AS of *FLM* pre-mRNA, resulting in the temperature-dependent production of functional *FLM-*β transcripts. Moreover, alterations in other splicing factors such as *ABA HYPERSENSITIVE1*/*CBP80* (*ABH1*/*CBP80*) and *STABILIZED1* (*STA1*) did not impact the *FLM-*β/*FLM-δ* ratio at different temperatures. Taken together, our data suggest that a temperature-dependent interaction between AtSF1 and *FLM* pre-mRNA controls flowering time in response to temperature fluctuations.

## Introduction

As sessile organisms, plants have evolved extensive developmental plasticity, allowing them to adjust their lifestyles in response to continuously varying environmental conditions (de Jong and Leyser, 2012). In particular, plants must sense and respond to environmental changes, such as temperature fluctuations, which can have major effects on growth and development. Even small changes in ambient temperature, for instance, can significantly affect the flowering time (Fitter and Fitter, 2002; Craufurd and Wheeler, 2009; Cook et al., 2012), with colder and warmer temperatures generally delaying and accelerating flowering, respectively.

Molecular genetic studies on Arabidopsis (*Arabidopsis thaliana*) have revealed that a variety of genes previously placed in different flowering pathways function in the ambient temperature pathway (Samach and Wigge, 2005; Lee et al., 2008; Penfield, 2008; McClung and Davis, 2010; Verhage et al., 2014; Capovilla et al., 2015b). Among them, MADS-box transcription factor genes such as *SHORT VEGETATIVE PHASE* (*SVP*) (Lee et al., 2007), *FLOWERING LOCUS M* (*FLM*) (Balasubramanian et al., 2006), and to a lesser extent *FLOWERING LOCUS C* (*FLC*), *MADS AFFECTING FLOWERING2* (*MAF2*), *MAF3*, *MAF4*, and *MAF5* (Gu et al., 2013), are known to be key players in the ambient temperature pathway. In particular, recent studies have shown that post-transcriptional mechanisms, such as the temperature-dependent stability of SVP protein (Lee et al., 2013) and alternative splicing (AS) of *FLM* (Pose et al., 2013) and *MAF2* (Airoldi et al., 2015) are important regulatory mechanisms at the molecular level within the ambient temperature pathway.

AS is when alternative splice sites are selected, resulting in the production of multiple mRNA isoforms from precursor-mRNA (pre-mRNA). Because splice site selection can be regulated by cell type, developmental stage, and environmental stimuli (Syed et al., 2012; Fu and Ares, 2014; Capovilla et al., 2018), AS plays a fundamental role in plant growth, development, and responses to external cues (Staiger and Brown, 2013; Filichkin et al., 2015). Recent studies suggest that AS of pre-mRNAs serves as a ‘molecular thermometer’ in the plant response to temperature perturbations (Capovilla et al., 2015a; Deng and Cao, 2017). One example of the role of temperature-dependent AS in temperature-responsive flowering is the regulation of *FLM*; mutually exclusive incorporation of the second or third exon leads to production of one of two predominant spliced isoforms, *FLM-*β and *FLM-δ*, at low and high ambient temperatures, respectively (Lee et al., 2013; Pose et al., 2013). Both the formation of distinct protein complexes between FLM-β/FLM-δ and SVP, and SVP protein stability at different temperatures contribute to floral transition in response to changes in ambient temperature. However, recent reports have revealed that the differential production of *FLM-*β transcripts at different temperatures is more important for controlling temperature-responsive flowering time (Lee et al., 2014; Lutz et al., 2015, 2017; Capovilla et al., 2017). Furthermore, high ambient temperature causes nonsense-mediated mRNA decay of aberrant *FLM* transcripts without a significant increase in *FLM-δ* transcript levels (Sureshkumar et al., 2016). Findings of these authors suggest that the contribution of *FLM-δ* isoform in the regulation of flowering may be minor.

Numerous studies have revealed that the changes in ambient temperature affect AS of splicing-related genes. RNA-sequencing data on two accessions of Arabidopsis and one genotype of *Brassica oleracea* ssp. *botrytis* showed a different splicing pattern of splicing-related genes upon ambient temperature changes (Verhage et al., 2017). In addition, different splicing patterns of *cyclin-dependent kinase* (*CDK*) *G1* in a temperature-dependent manner affects the AS of *AtU2AF65a* functioning in 3′ splice site recognition (Cavallari et al., 2018; Wang et al., 2019) and the lack of CDKG2/CYCLYN L1 complex leads to significantly different *FLM* splicing (Nicotra et al., 2010). Furthermore, the lesions in *AtGRP7*/*8* affecting the choice of alternative 5′ splice site in mRNA splicing result in changes in flowering time in a temperature-dependent manner (Steffen et al., 2019). These results suggest that splicing-related genes may be the target of temperature-dependent AS, thereby regulating plant growth and development in adaption to continuously changing temperatures.

Although we have previously shown that *FLM-*β transcripts are dramatically decreased in *atsf1-2* mutants (Lee et al., 2017), the molecular mechanism underlying *FLM* pre-mRNA processing by splicing factor(s) in the context of temperature-responsive flowering still remains unknown. Here, we demonstrate that *Arabidopsis splicing factor 1* (*AtSF1*) acting in 3’ splice-site recognition is responsible for ambient temperature-dependent AS of *FLM* pre-mRNA, resulting in the temperature-dependent production of functional *FLM-*β transcripts.

## Materials and Methods

### Plant Materials and Growth Conditions

All mutants of *Arabidopsis thaliana* used in this study are from the Columbia (Col-0) ecotype, unless otherwise noted. Seeds of wild-type accessions were obtained from the Arabidopsis Biological Resource Center (Alonso et al., 2003). *abh1-285*, *atsf1-2*, and *sta1-1* (Col-gl1) were described previously (Lee et al., 2006; Laubinger et al., 2008; Jang et al., 2014). Wild-type (Col-0), mutant, and transgenic plants were grown in soil or on Murashige and Skoog (MS) medium at 27, 23, or 16°C under long-day (LD) (16 h light/8 h dark) or short-day (SD) (8 h light/16 h dark) conditions at a light intensity of 120 μmol m^–2^ s^–1^. Flowering time was measured by scoring the total numbers of rosette and cauline leaves (total leaf number, TLN), or bolting days (BD), which were recorded when the primary inflorescence had reached a height of 0.5 cm; data are presented as box plots (Spitzer et al., 2014). In the box plots, center lines indicate the median, while plus signs (+) indicate the mean value; box limits show the 25th and 75th percentiles (lower and upper quartiles) as determined by the R software. Whiskers extend 1.5 times the interquartile range from the 25th and 75th percentiles, while outliers that exceed their whisker range are represented by ovals. To reveal statistical differences in flowering time, the data were analyzed using SPSS, version 24 (IBM SPSS Statistics). The number of plants counted is shown above each genotype in the box plot. The leaf number ratio (LNR, 16°C/23°C or 16°C/27°C) under LD and SD conditions was used as an indicator of temperature-responsive flowering (Lee et al., 2007). A hypothetical temperature-responsive plant produces a different total number of leaves at 23 and 16°C under LD conditions; thus, its LNR is closer to 2.0. Two reciprocal growth parameters [the rate of leaf production (leaves/day) and plastochron (days/leaf)] were also estimated based on ∼1–2 mm leaf primordia. For each plant, the number of leaf primordia visible by the naked eye was counted every day after germination until flowering initiation (Mendez-Vigo et al., 2010).

For vernalization, imbibed seeds were stored at 4°C for 4 weeks, then grown on soil or MS medium under SD conditions at 23°C. For gibberellic acid treatment, plants were grown on soil or MS medium under SD conditions at 23°C. After germination, plants were sprayed with 100 μM GA_3_ (Sigma-Aldrich, St. Louis, MI, United States) solution weekly until flowering.

### Expression Analyses

For RNA expression analysis, total RNA was extracted from whole seedlings using TRIzol reagent (Invitrogen, Carlsbad, CA, United States). Samples for reverse transcription polymerase chain reaction (RT–PCR) or real-time quantitative polymerase chain reaction (RT–qPCR) were harvested at Zeitgeber time (ZT) 8 and ZT 16 (under LD conditions) or ZT 4 and ZT 8 (under SD conditions), frozen immediately in liquid nitrogen, and then stored at −80°C until use. RNA quality was determined with a NanoDrop ND-2000 spectrophotometer (Thermo Scientific, Waltham, MA, United States), and only high-quality RNA samples (A260/A230 > 2.0 and A260/A280 > 1.8) were used for subsequent experiments. cDNA was synthesized from 1 μg of RNA per sample, using the Transcriptor First Strand cDNA Synthesis Kit (Roche Applied Science, Madison, WI, United States). RT–qPCR analysis, performed as described previously (Jang et al., 2014), was carried out in 384-well plates with a LightCycler 480 (Roche Applied Science) using Roche SYBR Green Master mixture (Roche Applied Science). A stably expressed gene (*PP2AA3*) was used as a reference gene. All RT–PCR or RT–qPCR experiments were carried out in three biological replicates (independently harvested samples) with three technical replicates each. The relative abundance of transcripts was determined as previously described (Lee et al., 2013). To reveal statistical differences in expression, the data were analyzed using SPSS, version 24 (IBM SPSS Statistics). The sequences of the oligonucleotides used in this study are listed in Supplementary Table 1.

For protein expression analysis, whole seedlings of *pAtSF1_964bp_::AtSF1:GUS atsf1-2* plants (Lee et al., 2017) were ground to a powder in liquid nitrogen, which was then suspended in a buffered solution of 50 mM Tris–HCl (pH 8.0), 150 mM NaCl, 10% glycerol, 0.5% Triton X-100 (Sigma-Aldrich), 2 mM phenylmethanesulfonyl fluoride, and complete protease inhibitor cocktail (Roche Applied Science). Protein concentrations were determined using Bradford solution (Bio-Rad, Hercules, CA, United States). Proteins were separated using 10% SDS-PAGE and transferred onto PVDF membranes (Bio-Rad) as described previously (Lee et al., 2017). PVDF membranes were then probed with a mouse monoclonal anti-GUS antibody (diluted 1:500; Santa Cruz Biotechnology, Dallas, TX, United States), followed by a secondary antibody (diluted 1:2000; Enzo Life Sciences, United Kingdom). Immunoreactive bands were visualized using an ECL detection reagent (Innotech, Daejeon, South Korea).

### RNA Electrophoretic Mobility-Shift Assay (EMSA)

The putative branch point sequence (BPS) of *FLM* was predicted using the online tool at http://www.cbs.dtu.dk/services/NetPGene/. RNA probes for the predicted BPS were synthesized by Integrated DNA Technologies (Coralville, IA, United States). A 20-mer RNA containing the human BPS and polypyrimidine (Py) tracts (5′-UAUACUAACAAUUUUUUUUU-3′) (Zhang et al., 2013) and RNAs containing the predicted BPS RNAs of *FLM* (BP1: 5′-UUCUAAUGCAUUUUUGUUUUAUCU-3′; BP2, 5′-UUCUAAUAUUCUUCUGGAUGCGGUUUUUGGUGUUAU-3′) were synthesized. Biotin was added to the 3′ ends of RNA probes using the RNA 3′ End Biotinylation Kit (Thermo Scientific). Because the full AtSF1 protein with His tag was not expressed under various conditions tested in this study, a truncated version of AtSF1^1–562^, containing the three domains of KH, Zinc Finger, and RNA recognition motif (RRM), was expressed with His tag in *Escherichia coli* and purified using the Ni-NTA Spin Kit (Qiagen, Hilden, Germany). RNA EMSA was performed using the LightShift^TM^ Chemiluminescent EMSA Kit (Thermo Scientific). Briefly, purified AtSF1 protein was incubated with biotin-labeled RNA probes at 23°C for 30 min. The reaction products were electrophoresed on 8% native polyacrylamide gels, transferred onto nylon membranes, and visualized as described in the manufacturer’s instructions.

### RNA Immunoprecipitation (RIP) Analysis

The RIP assay was conducted as described previously with minor modifications (Wierzbicki et al., 2008; Terzi and Simpson, 2009; Koster and Staiger, 2014). *pAtSF1_964bp_::AtSF1:GUS atsf1-2* seedlings (Lee et al., 2017) were grown on MS medium under temperature-shifted conditions; then, fresh plant samples were cross-linked in 1% formaldehyde using vacuum infiltration. After shearing of the chromatin via sonication, mouse monoclonal anti-GUS and c-Myc antibodies (Santa Cruz Biotechnology), and protein Agarose beads (Thermo Scientific) were added to the nuclear extract to immunoprecipitate the protein-RNA complexes. After eluting the protein-RNA complexes, DNA and proteins were removed by adding RQ1 RNase-free DNase (Promega, Wisconsin, United States) and proteinase K (Roche Applied Science). RNA fragments were isolated using acidic phenol:chloroform (Thermo Scientific), and RNAs were recovered by precipitation with ethanol. The immunoprecipitate or 10% of input RNA was subjected to qPCR. RIP experiments were carried out in three biological replicates (samples independently harvested on different days) with three technical triplicates each (RNA IP samples processed on the same day) and results were presented as a percentage of input (% input) (Livak and Schmittgen, 2001). Error bars indicate the standard error of the mean of the three biological replicates. All sequences of the oligonucleotides used in this study are listed in Supplementary Table 1.

## Results

### Mutation of *AtSF1* Leads to Ambient Temperature-Insensitive Flowering

**FIGURE 1:**
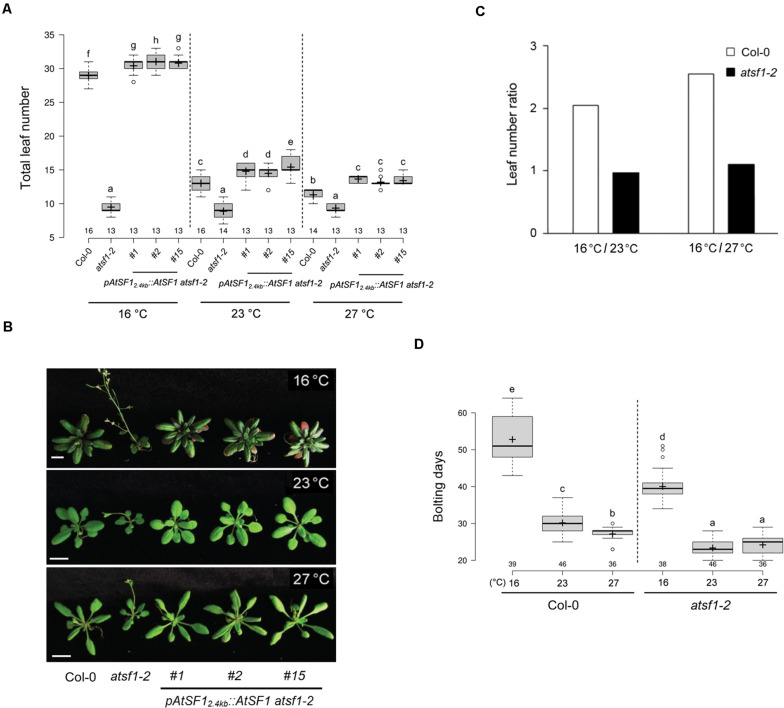
Flowering time phenotypes of *atsf1-2* mutants at different temperatures. **(A,B)** Box plot showing flowering time **(A)** and plant phenotypes **(B)** of wild-type (Col-0) plants, *atsf1-2* mutants, and rescued lines (*_*P*_AtSF1_2.4kb_::AtSF1 atsf1-2*) at 27, 23, and 16°C under LD conditions (see METHODS for further information about box plots). Error bars indicate the standard error of the mean of three biological replicates. Photographs were taken when *atsf1-2* mutants flowered. Scale bars, 1 cm. **(C)** Leaf number ratio (LNR, 16°C/23°C and 16°C/27°C) of wild-type plants and *atsf1-2* mutants (see METHODS for further information about LNR). **(D)** Box plot showing bolting days of wild-type plants and *atsf1-2* mutants grown at 27, 23, and 16°C under LD conditions. In **(A,D)**, letters indicate statistical groups determined with multiple comparisons with the Duncan method. Multiple comparisons were performed within temperatures and within genotypes. Groups were considered statistically different when *P* < 0.05.

A mutation in *AtSF1*, which contributes to 3′ splice-site recognition by binding directly to the intron BPS, leads to an altered flowering time at 23°C (Jang et al., 2014; Lee et al., 2017). We examined the flowering responses of this mutant at various temperatures (27, 23, and 16°C) under LD conditions. The *atsf1-2* mutants exhibited early flowering that was not substantially altered by changes in temperature [8.8, 9.9, and 9.6 leaves (total leaf number, TLN) at 27, 23, and 16°C, respectively] (Figures 1A,B and Supplementary Table 2). However, wild-type plants showed temperature-responsive flowering [11.9, 14.8, and 30.3 leaves (TLN) at 27, 23, and 16°C, respectively). Furthermore, leaf number ratio (LNR) of *atsf1-2* mutants were 1.0 and 1.1 (16°C/23°C and 16°C/27°C, respectively) (Figure 1C). However, LNR of wild-type plants were 2.0 and 2.5 (16°C/23°C and 16°C/27°C, respectively). This indicates that *atsf1-2* mutants did not respond to the changes in temperature, albeit they had slightly early flowering at 27°C (LNR of 16°C/27°C = 1.1). The temperature insensitivity of the *atsf1-2* mutants was also observed under SD conditions (Supplementary Figures 1A,B and Supplementary Table 2), albeit flowering time of the *atsf1-2* mutants was slightly different. The LNR of *atsf1-2* mutants were 1.1 and 1.7 at 16°C/23°C and 16°C/27°C, respectively (cf. wild-type plants = 1.5 and 4.2, Supplementary Figure 1C). However, the ambient temperature-insensitive flowering of *atsf1-2* mutants was completely suppressed in three independent *pAtSF1_2.4kb_::AtSF1 atsf1-2* plants (Lee et al., 2017), irrespective of light conditions (Figures 1A,B, Supplementary Figures 1A,B, and Supplementary Table 2), which indicates that the altered activities of *AtSF1* affect temperature-responsive flowering. Because *atsf1-2* mutants show a severe dwarf phenotype, its ambient temperature-insensitive flowering phenotype may result from slower growth rate, compared with the wild-type plants. Therefore, we also checked the bolting days (BD) at different temperatures under LD and SD conditions. The BD of *atsf1-2* mutants were earlier than those of wild-type plants at different temperatures (Figure 1D and Supplementary Figure 1D). For instance, the BD of *atsf1-2* mutants at different temperatures under LD conditions were 40.1, 23.0, and 24.3 at 16, 23, and 27°C, respectively (c.f. wild-type plants = 52.9, 30.2, and 27.2). The earlier BD in *atsf1-2* mutants were also observed at 16 and 23°C under SD conditions, albeit the effects were more pronounced in SD than in LD. These results indicate that the differences in BD between wild-type plants and *atsf1-2* mutants at indicated temperatures (cf. *atsf1-2* mutants = 1.7 at both 16°C/23°C and 16°C/27°C under LD conditions, wild-type plants = 1.9 at both 16°C/23°C and 16°C/27°C under LD conditions) were smaller than those in TLN between them, irrespective of light conditions. Furthermore, the average rate of plastochron in *atsf1-2* mutants was much higher than that of wild-type plants at both temperatures (cf. *atsf1-2* mutants = 5.4 and 6.6 at 23 and 16°C, respectively, and wild-type plants = 3.8 and 3.4 at 23 and 16°C, respectively), although the average rate of leaf production between the mutants and wild-type plants was similar (cf. *atsf1-2* mutants = 0.2 at both 16 and 23°C and wild-type plants = 0.3 at both 16 and 23°C, Supplementary Figure 2). These suggest that the early flowering of *atsf1-2* mutants may also be due to defects in development such as the regulation of plastochron length. However, other floral inductive conditions, such as vernalization and gibberellic acid treatments, did not affect *AtSF1* expression and both the *atsf1-2* mutants and wild-type plants responded similarly, to both conditions (Supplementary Figure 3). Considering the effects of *atsf1* mutation on both leaf number and the rate of vegetative growth at different temperatures, these data suggest that alteration of *AtSF1* activity affects temperature-responsive flowering.

### AtSF1 Negatively Regulates the Expression of Floral Repressors

**FIGURE 2:**
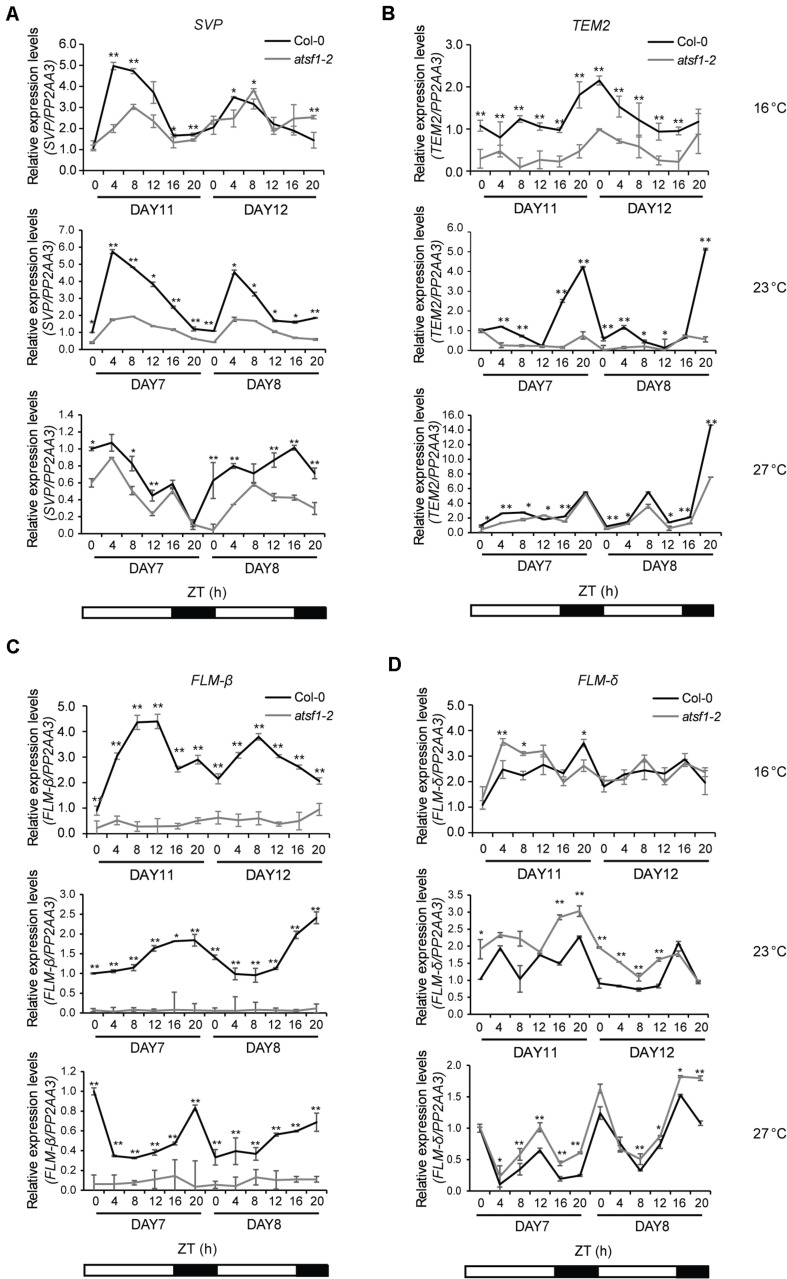
Diurnal expression patterns of *SVP, TEM2, FLM-*β, and *FLM-δ* in the *atsf1-2* mutant at different temperatures. Total RNA was sampled at 4-h intervals from 7- to 8-day-old (27 and 23°C) or from 11- to 12-day-old (16°C) seedlings grown at the indicated temperatures under LD conditions, and the expression of *SVP* **(A)**, *TEM2* **(B)**, *FLM-*β **(C)**, and *FLM-δ* **(D)** was measured by RT–qPCR (Student’s *t*-test, *P < 0.05; **P < 0.01). Expression levels in wild-type (Col-0) plants at ZT 0 on day 7 or 11 at the indicated temperatures were defined as 1.0. Error bars indicate the standard error of the mean of three biological replicates. The *PP2AA3* (*AT1G13320*) gene was used as an internal control.

Because *atsf1-2* mutants showed early flowering phenotypes at different temperatures (Figure 1, Supplementary Figure 1 and Supplementary Table 2), we measured the expression of known ambient temperature pathway genes that repress flowering over approximately a 2-day diurnal time course in *atsf1-2* and wild-type plants in similar developmental stages grown at 16, 23, or 27°C under LD conditions. In *atsf1-2* mutants the expression of *SVP* and *TEMPRANILLO2* (*TEM2*) (Marin-Gonzalez et al., 2015), important floral repressors in the ambient temperature pathway, was reduced at most temperatures, albeit reduced *SVP* expression in *atsf1-2* mutants was observed only on day 11 at 16°C and unaltered *TEM2* expression in *atsf1* mutant background was found only at 27°C (Figures 2A,B). Furthermore, the diurnal expression patterns of *FLM-*β were almost abolished at all temperatures in *atsf1-2* mutants, whereas the expression of *FLM-δ*, another spliced isoform of *FLM*, marginally increased (Figures 2C,D). The reduced expression patterns of these floral repressors were also observed in *atsf1-2* mutants under SD conditions, although the expression of *FLM-δ* was significantly altered only at 16°C (Supplementary Figure 4). The expression of a major floral repressor, *FLC*, was previously known to be roughly unaffected in the *atsf1-2* mutants (Jang et al., 2014). These results indicate that the temperature-insensitive flowering phenotype of *atsf1-2* mutants is a result of defects in floral repression in response to temperature changes.

### Mutation of *AtSF1* Affects the Expression and Intron Splicing of *FLM*

**FIGURE 3:**
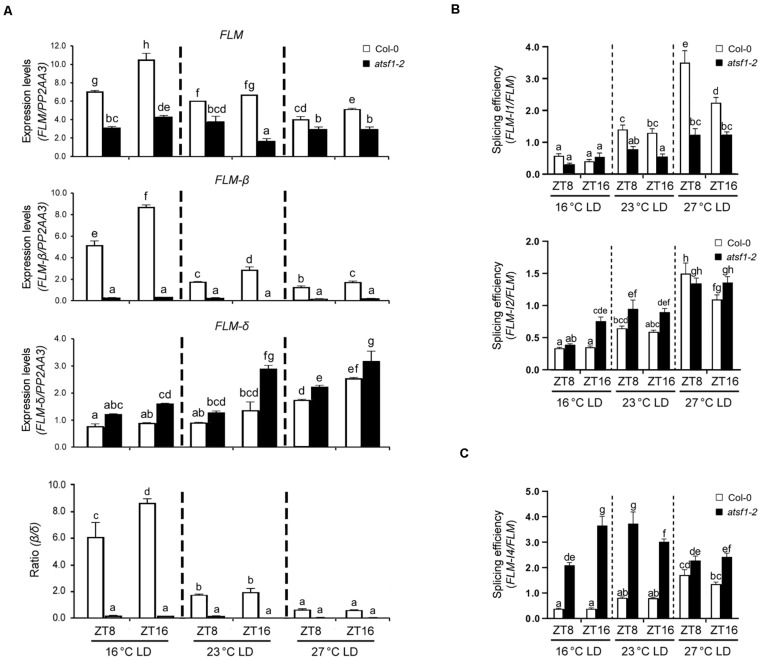
Alternative splicing patterns of *FLM* in *atsf1-2* mutants. **(A)** The expression of *FLM*, *FLM-*β, and *FLM-δ* isoforms in *atsf1-2* mutants at different temperatures. In 8-day-old (27 and 23°C) or 12-day-old (16°C) seedlings under LD conditions, RT–qPCR was performed at the indicated temperatures and time points (ZT 8 and 16). Expression levels were normalized to *PP2AA3* gene. Error bars indicate the standard error of the mean of three biological replicates. **(B,C)** The splicing efficiency of introns 1 and 2 **(B)**, and intron 4 **(C)**. The splicing efficiency was calculated as the ratio of spliced vs. unspliced *FLM* transcripts. In **(A–C)**, statistical analysis was performed as described in Figure 1.

We found that *FLM* expression is markedly reduced in *atsf1-2* mutants and that the *atsf1* mutation greatly changes the relative ratio of *FLM-*β and *FLM-δ* transcripts (Figure 2 and Supplementary Figure 4; Lee et al., 2017). Recent studies have also shown that the temperature-dependent AS of *FLM* contributes to temperature-responsive flowering (Lee et al., 2013; Pose et al., 2013; Steffen et al., 2019). This led us to investigate the specific effects of the temperature-dependent AS of *FLM* by AtSF1. We measured the overall levels of total *FLM* transcripts, as well as the individual levels of the two *FLM* splice isoforms (*FLM-*β and *FLM-δ*), at different temperatures. The overall *FLM* transcript levels were markedly reduced in *atsf1-2* mutants at all temperatures (Figure 3A), albeit the reduction of the total *FLM* transcript levels was lesser at 23 and 27°C than at 16°C compared with that of wild-type plants. The difference in total *FLM* expression between wild-type plants and *atsf1-2* mutants decreased with increasing temperature (approximately 2. 3-, 1. 7-, and 1.5-fold at 16, 23, and 27°C, respectively). Consistently with previous results (Lee et al., 2013, 2014; Pose et al., 2013), in wild-type plants the expression of *FLM-*β increased at 16°C, whereas *FLM-δ* levels increased at 27°C (Figure 3A). However, in *atsf1-2* mutants, *FLM-*β transcript levels dramatically decreased (approximately 20. 4-, 10. 4-, and 7.1-fold at 16, 23, and 27°C, respectively), whereas those of *FLM-δ* increased marginally (approximately 1. 7-, 1. 4-, and 1.3-fold at 16, 23, and 27°C, respectively) at all temperatures compared with wild-type plants (Figure 3A). Furthermore, the average *FLM-*β/*FLM-δ* ratio in wild-type plants decreased with increasing temperature (7.4, 1.9, and 0.7 at 16, 23, and 27°C, respectively), whereas that in *atsf1-2* mutants was not changed (Figure 3A). The expression patterns of total *FLM* transcripts and the two *FLM* splicing isoforms observed under LD conditions in *atsf1-2* mutants at different temperatures were also observed under SD conditions (Supplementary Figure 5), albeit the increased levels of *FLM-δ* were not apparent in *atsf1-2* mutants at 27°C. Together with the down-regulation of *TEM2* and *SVP* in *atsf1-2* mutants at most temperatures (Figure 2 and Supplementary Figure 4), these results indicate that the marked change in *FLM-*β as a functional repressor form in *atsf1-2* mutants may lead to an early flowering phenotype at all temperatures.

Because *AtSF1* mutations significantly affect RNA splicing, especially for intron excision (Zhu et al., 2020), we analyzed the expression of all *FLM* mRNAs retaining introns 1 and 2 at 16 and 23°C. We found that the expression levels of *FLM* intron 1 and 2 retention forms were altered in *atsf1-2* mutants at all temperatures (Supplementary Figures 6A,B), which leads to a significant change in the splicing in *atsf1-2* mutants (Figure 3B). We further examined the levels of *FLM* intron 4 retention form in *atsf1-2* mutants at different temperatures. Retention forms of the in frame intron 4, either in combination with exon 2 or 3, could make translated proteins (Nibau et al., 2019). We observed the increased levels of *FLM* intron 4 retention forms in *atsf1-2* mutants at 16 and 23°C, and a significant increase in the splicing (Supplementary Figure 6A,B and Figure 3C). We also detected other *FLM* intron retention forms in *atsf1-2* mutants (Supplementary Figure 7). Taken together, these data suggest that the changes in *FLM* splicing in *atsf1-2* mutants lead to alteration in the levels of mature *FLM* transcripts, which may affect flowering phenotype of *atsf1-2* mutants.

### Changes in Temperature do Not Affect *AtSF1* RNA or AtSF1 Protein Expression

**FIGURE 4:**
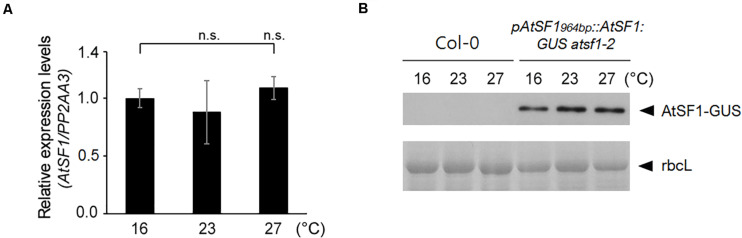
*AtSF1* RNA and AtSF1 protein expression under shifted temperature conditions. **(A)** RNA expression of endogenous *AtSF1* in 9-day-old wild-type (Col-0) seedlings at different temperatures after one day shift from 23 to 16 or 27°C. Samples were harvested at ZT 16 under LD conditions. Expression levels at 16°C were defined as 1.0. Error bars indicate the standard error of the mean of the three biological replicates. **(B)** Abundance of AtSF1-GUS protein in 9-day-old *pAtSF1_964bp_::AtSF1:GUS atsf1-2* plants grown at different temperatures after one day shift from 23 to 16°C or 27°C. Samples were harvested at ZT 16 under LD conditions. Ponceau S-stained Rubisco large subunit (rbcL) was used as a loading control.

To determine whether AtSF1 activity might be temperature-dependent, we first asked whether *AtSF1* RNA expression is regulated by temperature. Specifically, we performed RT–qPCR on RNA after a shift of 8-day-old wild-type seedlings from 23 to 16°C or 27°C. *AtSF1* expression was unaltered under the different temperature conditions (Figure 4A). We also monitored the AtSF1-GUS protein accumulation in 8-day-old *pAtSF1_964bp_::AtSF1:GUS atsf1-2* seedlings after a shift from 23 to 16°C or 27°C (Lee et al., 2017). AtSF1-GUS protein levels remained unchanged under the different temperature conditions (Figure 4B). Together with a previous report that the alternative spliced transcripts of *AtSF1* were not found in wild-type plants under shifted temperature conditions (Verhage et al., 2017), these results suggest that *AtSF1* RNA expression and AtSF1 protein stability are not differentially regulated with temperature fluctuations. Therefore, it is likely that AtSF1 activity may be regulated by different mechanisms as per the requirements of the temperature-dependent AS of *FLM* pre-mRNA.

### *FLM* Is a Target Transcript of AtSF1 *in vitro* and *in vivo*

Because the expression changes in *FLM-*β and *FLM-δ* isoforms were observed in *atsf1-2* mutants (Figures 2, 3 and Supplementary Figures 4, 5), and the levels of *AtSF1* RNA and AtSF1 protein were unaltered at different temperatures (Figure 4), the changes in temperature may affect the AS of *FLM* pre-mRNA by modulating the physical interaction between AtSF1 and *FLM* pre-mRNA. To explore this possibility, we performed EMSAs to investigate the *in vitro* RNA-binding properties of AtSF1. AtSF1 bound to the putative AtSF1-binding sequences (FLM BP1 and FLM BP2) in the BPS and Py tract in introns 1 and 2 of *FLM* pre-mRNA, which was predicted by an *in silico* search (Supplementary Figures 8, 9). The RNA probes (FLM BP1 and FLM BP2) used for EMSA analysis also included the BPS consensus sequence containing 5′-CU(U/A)AU-3′ required for AtSF1’s binding (Zhu et al., 2020). Moreover, competition assays showed that the addition of excess amounts of unlabeled RNA competitor probes to the EMSAs greatly reduced the intensities of the respective shifted bands (Supplementary Figure 9). These results indicate that AtSF1 binds to the BPS sites of introns 1 and 2 of *FLM* pre-mRNA *in vitro*.

**FIGURE 5:**
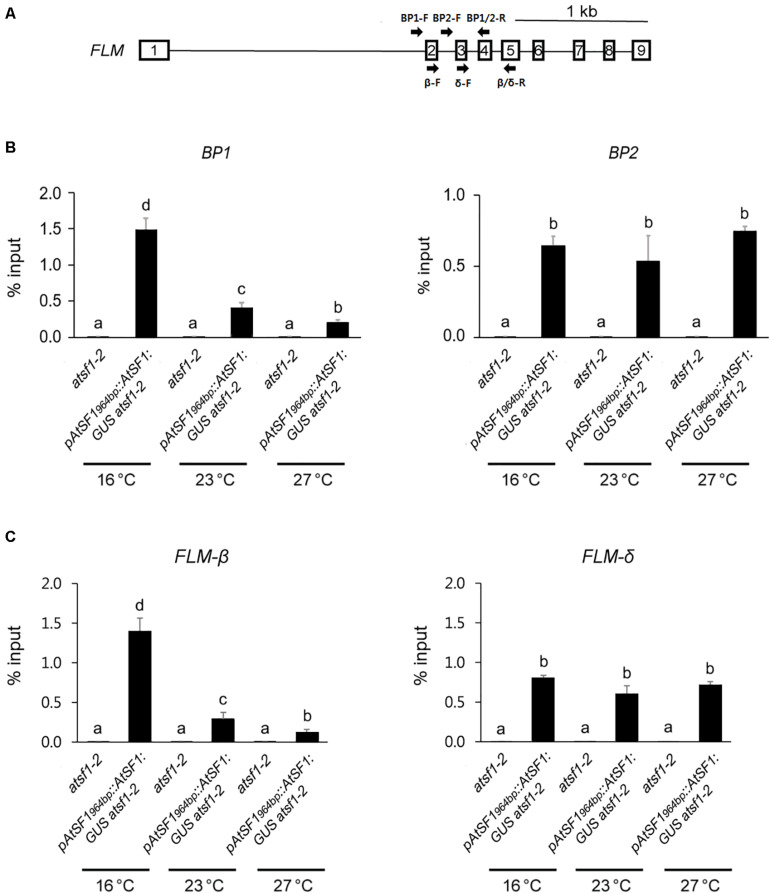
*FLM* is an *in vivo* target of AtSF1. Schematic diagram shows the positions of primers used for the RIP assay. **(A)** Schematic representation of the *FLM* locus. Square boxes and lines represent exons and introns, respectively. Arrows indicate primers used for RIP assay. BP1 (BP1-F and BP1/2-R) and BP2 primer (BP2-F and BP1/2-R) sets amplify intron 1-specific transcripts and intron 2-specific transcripts, respectively. *FLM-*β (β-F and β/δ-R) and *FLM-δ* primer (δ-F and β/δ-R) sets amplify exons 2/4/5 and exons 3/4/5, respectively. **(B,C)** The differential binding of AtSF1-GUS to BP1 or BP2, and *FLM-*β or *FLM-δ* transcripts. For RIP analysis of AtSF1-GUS binding to *FLM* pre-mRNA using anti GUS antibody, 9-day-old seedlings of *pAtSF1_964bp_::AtSF1:GUS atsf1-2* and *atsf1-2* plants at different temperatures under LD conditions (ZT 16) after one day shift from 23 to 16 or 27°C were used. The abundance of differently spliced *FLM* transcripts was quantified by qPCR. Error bars indicate the standard error of the mean of three biological replicates. In panels **(B,C)**, statistical analysis was performed as described in Figure 1.

We next investigated whether AtSF1 associates with the *FLM* pre-mRNA transcripts *in vivo* in order to regulate its pre-mRNA splicing. To this end, we performed RNA immunoprecipitation (RIP) assays using 9-day-old *pAtSF1_964bp_::AtSF1:GUS atsf1-2* plants and *atsf1-2* mutants after a shift from 23 to 16°C or 27°C under LD conditions. RIP assays using anti GUS antibody with primer sets amplifying intron 1-exons 2/3/4 or intron 2-exons 3/4 revealed that AtSF1-GUS efficiently co-immunoprecipitated BP1 and BP2 at different temperatures (Figures 5A,B). Interestingly, AtSF1-GUS binding to BP1 was 3.7-fold higher at 16°C than at 23°C, whereas its binding to BP1 was 0.5-fold lower at 27°C than at 23°C. However, AtSF1-GUS binding to BP2 was not significantly changed at different temperatures (1.2-fold and 1.4-fold at 16°C vs. 23°C and 27°C vs. 23°C, respectively). We also observed the preferential binding of AtSF1-GUS to exon regions of *FLM* at different temperatures in a RIP assay using GUS antibody, with primer sets amplifying exons 2/3/4/5 within *FLM-*β (4.8-fold and 0.4-fold at 16°C vs. 23°C and 27°C vs. 23°C, respectively) (Figures 5A,C). However, the binding of AtSF1-GUS to exon regions (exons 3/4/5) within *FLM-δ* was not significantly altered (1.3-fold and 1.2-fold at 16°C vs. 23°C and 27°C vs. 23°C, respectively). For the control RIP experiment using anti c-Myc antibody, we did not find significant changes in AtSF1-GUS binding to BP1, BP2, *FLM-*β, and *FLM-δ* regions (Supplementary Figure 10). These results indicate that AtSF1 preferentially binds to *FLM-*β transcripts at a lower temperature.

To examine whether the preferential binding of AtSF1 to *FLM-*β transcripts at 16°C is caused by the higher abundance of *FLM* pre-mRNA at 16°C, we checked the expression levels of *FLM* pre-mRNA after a shift from 23 to 16°C or 27°C. Under shifted temperature conditions, *FLM* pre-mRNA expression was not significantly altered (Supplementary Figure 11). Taken together, these results suggest that *FLM* is an *in vivo* target of AtSF1 and that AtSF1 associates with *FLM* pre-mRNA *in vivo* to preferentially produce functional *FLM-*β transcripts in a temperature-dependent manner.

### Effect of Other Splicing Factors on Temperature-Responsive Flowering

**FIGURE 6:**
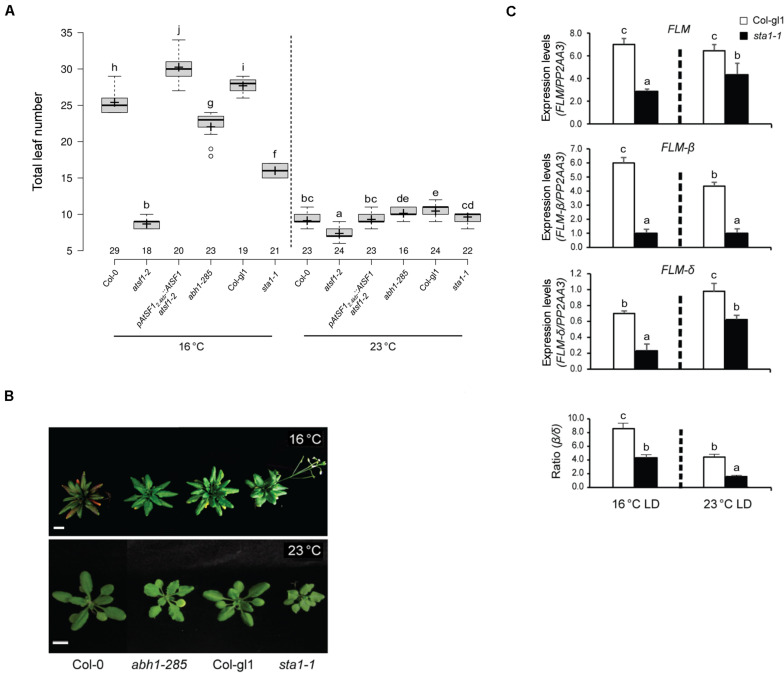
Flowering time phenotypes of other splicing factor mutants at different temperatures. Box plot of flowering times **(A)** (see section “Materials and Methods” for further information on box plots) and phenotypes **(B)** of plants of the indicated genotypes grown at 23 and 16°C under LD conditions. Error bars indicate the standard error of the mean of three biological replicates. Photographs were captured when *sta1-1* mutants flowered (Scale bars, 1 cm). **(C)** Expression levels of *FLM*, *FLM-*β, and *FLM-δ* transcripts in *sta1-1* mutants, measured by RT–qPCR, at the indicated temperatures and time points (ZT 8 and 16) (Student’s *t*-test; **P < 0.01). Expression levels were normalized to the *PP2AA3* gene. The *FLM-*β/*FLM-δ* ratio is shown below. Error bars indicate the standard error of the mean of three biological replicates. In panels **(A,C)**, statistical analysis was performed as described in Figure 1.

Mutations in *ABA HYPERSENSITIVE1*/*CBP80* (*ABH1*/*CBP80*), a cap binding protein, and *STABILIZED1* (*STA1*), a putative component of the U5 snRNP complex involved in the second step of splicing, affect flowering (Lee et al., 2006; Kuhn et al., 2007). Hence, we analyzed the temperature responses of *abh1-285* and *sta1-1* mutants at 23 and 16°C under LD conditions. The *abh1-285* mutants exhibited a temperature response similar to that of wild-type plants, whereas *sta1-1* mutants had an attenuated temperature response (Figures 6A,B and Supplementary Table 2), raising the possibility that *STA1* affects the AS of *FLM* pre-mRNA. However, the expression levels of total *FLM*, *FLM-*β, and *FLM-δ* in *sta1-1* mutants decreased at both temperatures (Figure 6C), explaining the early flowering of *sta1-1* mutants. Moreover, a decrease in the *FLM-*β/*FLM-δ* ratio with increasing temperature, similar to that in wild-type plants (8.5- and 4.4-fold at 16 and 23°C, respectively), was observed in *sta1-1* mutants (4.4- and 1.7-fold at 16 and 23°C, respectively), indicating that *sta1* mutation does not affect the AS of *FLM* pre-mRNA at different temperatures. These results suggest that AtSF1 exerts distinct responses to different temperatures by differentially affecting the *FLM* pre-mRNA splicing.

## Discussion

Recent reports have revealed that the expression levels of *FLM-*β transcripts explain much of the natural variation in flowering time (Lutz et al., 2015, 2017; Capovilla et al., 2017) and the AS of pre-mRNAs functions as a “molecular thermometer” in response to temperature fluctuations in plants (Capovilla et al., 2015b; Deng and Cao, 2017). However, little is known about the regulation of flowering time by splicing factor(s) in response to changes in ambient temperature. In this study, we show that AtSF1 is a splicing factor for the temperature-dependent splicing of *FLM* pre-mRNA in temperature-responsive flowering in *Arabidopsis*.

### Vegetative Developmental Defects Caused by *AtSF1* Mutation Partly Affects the Regulation of Flowering Time

In plants, environmentally or genetically induced changes in flowering time correlate with the number of leaves produced during vegetative development (Martinez-Zapater and Somerville, 1990; Koornneef et al., 1991). Our previous reports have shown that *atsf1-2* mutants have several developmental defects such as flowering time, leaf size, and plant height as well as altered heat stress and abscisic acid (ABA) responses (Jang et al., 2014; Lee et al., 2017). In this study, we focused on flowering time of *atsf1-2* mutants at different ambient temperatures and we found that *AtSF1* mutations resulted in temperature-insensitive flowering (Figure 1 and Supplementary Figure 1). Because *atsf1-2* mutants had a severe dwarf phenotype, this developmental defects may lead to its temperature-insensitive flowering phenotype. This notion is supported by several lines of evidence. First, the differences in TLN between wild-type plants and *atsf1-2* mutants at different temperatures were larger than those in BD between them (Figure 1 and Supplementary Figure 1). For instance, *atsf1-2* mutants flowered 24.2% earlier than wild-type plants at 16°C under LD conditions but it produced 68.3% less leaves. Second, *atsf1-2* mutants had a highly reduced plastochron index at different temperatures compared with wild-type plants (Supplementary Figure 2). These findings suggest that vegetative developmental defects observed in *atsf1-2* mutants partly cause its temperature-insensitive flowering phenotype. Given that the fact that *microRNA156* (*miR156*) and its targeted *SQUAMOSA PROMOTER BINDING PROTEIN-LIKE* (*SPL*) genes are involved in plastochron length and flowering time (Wang et al., 2008), further investigation is needed to elucidate the role of AtSF1 in the regulation of plastochron length.

### Floral Repressors Such as *FLM*, *SVP*, and *TEM2* Are AtSF1’s Targets in the Temperature-Dependent Flowering

Mutations in splicing-related genes are known to affect pre-mRNA splicing or mRNA levels of a specific set of genes (Wang et al., 2012; Xin et al., 2017; Capovilla et al., 2018). We have also noted altered splicing of only a few genes in the *atsf1-2* mutants (Jang et al., 2014), which suggests that aberrantly spliced transcripts may be rapidly removed by RNA degradation systems (Drechsel et al., 2013). This notion is supported by the observation that splicing failure leads to a general reduction in mRNA transcripts without corresponding accumulation of unspliced pre-mRNA (Houseley and Tollervey, 2009). Here, we showed that AtSF1 affects flowering time in the ambient temperature pathway and regulates splicing of *FLM* pre-mRNA in a temperature-dependent manner. This notion is supported by several lines of evidence. First, *atsf1-2* mutants showed temperature-insensitive flowering at different temperatures under LD and SD conditions, whereas the flowering of rescued lines was similar to that of wild-type plants (Figure 1, Supplementary Figure 1, and Supplementary Table 2). Second, the early flowering phenotype of *atsf1-2* mutants at different temperatures was due to the down-regulation of *SVP*, *FLM*, and *TEM2* as important repressors acting within the ambient temperature pathway (Figure 2 and Supplementary Figure 4). Third, the marked down-regulation in *FLM-*β was observed in the *atsf1-2* mutants at different temperatures (Figure 3 and Supplementary Figure 5). Lastly, RIP assays in *pAtSF1_964bp_::AtSF1:GUS atsf1-2* plants under shifted temperature conditions revealed that AtSF1-GUS directly binds to *FLM* pre-mRNA, thereby affecting the production of functional *FLM-*β transcripts at different temperatures (Figure 5 and Supplementary Figure 10). These findings suggest that the reduced expression of floral repressors like *SVP* and *TEM2* could explain the temperature-insensitive flowering of *atsf1* mutants at most temperatures, and *FLM* is likely one of AtSF1’s splicing targets in the ambient temperature pathway. However, given that lesions in spliceosomal components alter pre-mRNAs’ splicing at a large scale (Marquez et al., 2012; Reddy et al., 2013; Klepikova et al., 2016) and *AtSF1* mutation affects the production of intron retention forms of *FLM* (Figure 3 and Supplementary Figures 6, 7), we cannot exclude the possibility that alteration of pre-mRNA splicing of *FLM* in *atsf1-2* mutants may be a possible cause for the development of the temperature-insensitive flowering phenotype; rather, the defects in pre-mRNA splicing or expression of multiple genes in the regulation of temperature-responsive flowering contribute to the phenotype of *atsf1-2* mutants. This notion is supported by the observations that *SVP* and *TEM2* expression significantly reduced in *atsf1-2* mutants (Figure 2 and Supplementary Figure 4), and *AtSF1* mutation affects the splicing patterns of many genes involved in chloroplast development under cold stress (Zhu et al., 2020). Thus, further investigation is required to elucidate the mechanisms between AtSF1 and its targets by RIP-Seq or cross-linking immunoprecipitation-Seq for genome-wide scale analysis.

An important question is whether the binding of AtSF1 to *FLM* pre-mRNA is also involved in the production of *FLM-δ* transcripts. The possibility of such a binding is supported by the observations that the levels of *FLM-δ* transcripts were marginally higher in the *atsf1-2* mutants (Figure 3 and Supplementary Figure 5) and that AtSF1 bound to FLM BP2 RNA sequences including the putative BPS and Py tract *in vitro* (Supplementary Figures 8, 9). However, *in vivo* RIP assays did not show the preferential binding of AtSF1 to intron 2-exons 3/4 or exons 3/4/5 at different temperatures (Figure 5). Furthermore, the deletion of RNP1/2 within the RNA recognition motif (RRM) of AtSF1 results in slightly increased *FLM-*β transcripts with unaltered levels of *FLM-δ* transcripts (Lee et al., 2017). These findings suggest that the formation of *FLM-δ* transcripts does not fully require AtSF1 function and that other splicing factors need to be involved in the production of *FLM-δ* transcripts.

### Relationship Between AtSF1 and Other Splicing Factors in Splicing of *FLM* pre-mRNA

It is likely that there are other splicing factors involved in the temperature-dependent AS of *FLM* pre-mRNA in addition to AtSF1, as shown by recent reports (Park et al., 2019; Steffen et al., 2019). For instance, loss of *AtU2AF65a* and *AtU2AF65b* activities leads to altered levels of *FLM-*β or *FLM-δ* transcripts, although the marked changes in *FLC* expression in *atu2af65a* or *atu2af65b* mutants could be the main cause of its flowering phenotype (Park et al., 2019; Xiong et al., 2019). We have previously shown that AtSF1 interacts with AtU2AF65 proteins (Jang et al., 2014). This may lead to a speculation that AtSF1 together with AtU2AF65 may control temperature-responsive flowering by regulating the AS of *FLM* pre-mRNA. However, neither *atu2af65a* nor *atu2af65b* mutant show any impairment in temperature-responsive flowering (Park et al., 2019). Furthermore, the *atu2af65a* mutants show late flowering, whereas the *atu2afF65b* mutants exhibit early flowering like the *atsf1-2* mutants. This phenotypic discrepancy among these mutants abolishes our speculation on their co-operation in temperature responsive flowering. This discrepancy has to be explored further in order to understand their relationships in flowering time control. Furthermore, a recent report shows that a CDKG2 (kinase)/CYCL1 complex affects the AS of *FLM* pre-mRNA in a temperature dependent manner contributing to temperature-responsive flowering, suggesting that phosphorylation of splicing factors may play a role in the AS of *FLM* pre-mRNA (Nibau et al., 2019). The phosphorylation of human SF1 enhances its interaction with U2AF65 (Wang et al., 2013; Chatrikhi et al., 2016). The phosphorylation status dynamics of AtSF1 protein are yet unknown, but further investigation on which splicing factors and their combinations control the levels of canonical *FLM* spliced isoforms would provide a better understanding of flowering behavior at different ambient temperatures.

The altered levels of *AtGRP7* and *AtGRP8* also affect AS of *FLM* pre-mRNA (Steffen et al., 2019). Given that loss of *AtGRP7* together with a reduction of *AtGRP8* (*atgrp 7-1 8i* mutants) shows significant late flowering phenotype under SD conditions at 20 and 27°C, but not at 16°C, it is likely that AtGRP7/8 cannot work together with AtSF1 to control flowering time. However, it is interesting that *FLM-*β expression in *atgrp 7-1 8i* mutants decreases marginally at different temperatures, whereas *FLM-δ* expression increases markedly (Steffen et al., 2019). The *atsf1-2* mutants show the opposite *FLM* expression pattern, in which the *FLM-δ* expression increases only marginally but *FLM-*β expression is almost abolished (Figure 3 and Supplementary Figure 5). These results suggest that these proteins may work differently on the AS of *FLM* pre-mRNA, although *FLM-δ* transcripts seem to have no effect to the regulation of temperature-responsive flowering (Sureshkumar et al., 2016; Capovilla et al., 2017; Lutz et al., 2017). Further identification of splicing factors affecting the levels of *FLM-*β transcripts will facilitate studies aimed at determining whether these splicing factors interact with AtSF1 to preferentially produce *FLM-*β isoforms.

### Role of AtSF1 in the Regulation of Temperature-Responsive Flowering

Here, we identified the preferential binding of AtSF1 to *FLM* pre-mRNA at cooler temperatures (16°C), which could explain the role of *FLM-*β transcripts for the regulation of temperature-responsive flowering. That is, we show the direct binding of the AtSF1 protein to the BPS of *FLM* pre-mRNA intron 1, preferentially producing *FLM-*β under cooler temperatures (Figure 5 and Supplementary Figure 10). Our results propose that the temperature-dependent binding of AtSF1 to *FLM* pre-mRNA results in the production of major functional *FLM-*β transcripts, which in turn affect temperature-responsive flowering (Figure 1 and Supplementary Figures 1, 2). The cooler ambient temperature favors the binding of AtSF1 to the BPS in intron 1 of *FLM* pre-mRNA, yielding the functional *FLM-*β isoform, leading to the formation of the SVP–FLM-β complex that represses flowering by binding its complex to the genomic regions of the floral activators. At a warmer ambient temperature (27°C), the binding of AtSF1 to the BPS in intron 1 of *FLM* pre-mRNA is reduced significantly. This leads to decreased levels of the SVP–FLM-β complex and reduced SVP stability, resulting in flowering at this temperature.

It has been suggested that *FLM* pre-mRNA AS may play a role as a molecular thermometer in controlling the flowering time according to the change in ambient temperature (Capovilla et al., 2015b; Deng and Cao, 2017). This study revealed that AtSF1 can play a key role in this AS process. We have previously revealed that the RRM domain of AtSF1 protein is required for the formation of full *FLM-*β transcript level (Lee et al., 2017). The mutant line rescued by the RRM domain deletion AtSF1 construct (*pAtSF1_2.4kb_::AtSF1ΔRRM:GUS atsf1-2*) recovered almost all other developmental defects except flowering time (Lee et al., 2017). So, this would be a useful research tool in the clear identification of AtSF1’s role in the link between the AS of *FLM* pre-mRNA and flowering activators in temperature-dependent flowering control.

Taken together, we propose that the temperature-dependent splicing of pre-mRNAs of a specific set of genes by splicing factor(s) fine-tunes immediately temperature-responsive flowering over the plant’s life cycle and the temperature-dependent AS of *FLM* pre-mRNA regulated by AtSF1 could be one candidate for that mechanism.

## Data Availability Statement

The original contributions presented in the study are included in the article/Supplementary Material, further inquiries can be directed to the corresponding author/s.

## Author Contributions

JHL and J-KK conceived and designed the research. KL, KC, HL, and J-HP conducted the experiments. KL, JHL, and J-KK analyzed the data. KL, JHL, and J-KK wrote the manuscript. All authors read and approved the manuscript.

## Conflict of Interest

The authors declare that the research was conducted in the absence of any commercial or financial relationships that could be construed as a potential conflict of interest.
