# Supplementary material for: Role of Arabidopsis Splicing factor SF1 in Temperature-Responsive Alternative Splicing of FLM pre-mRNA
Source: Front Plant Sci. 2020 Dec 1;11:596354. doi: 10.3389/fpls.2020.596354 (PMC7735993; doi:10.3389/fpls.2020.596354)
Supplement: Supplementary file 1 [file Data_Sheet_1.docx]

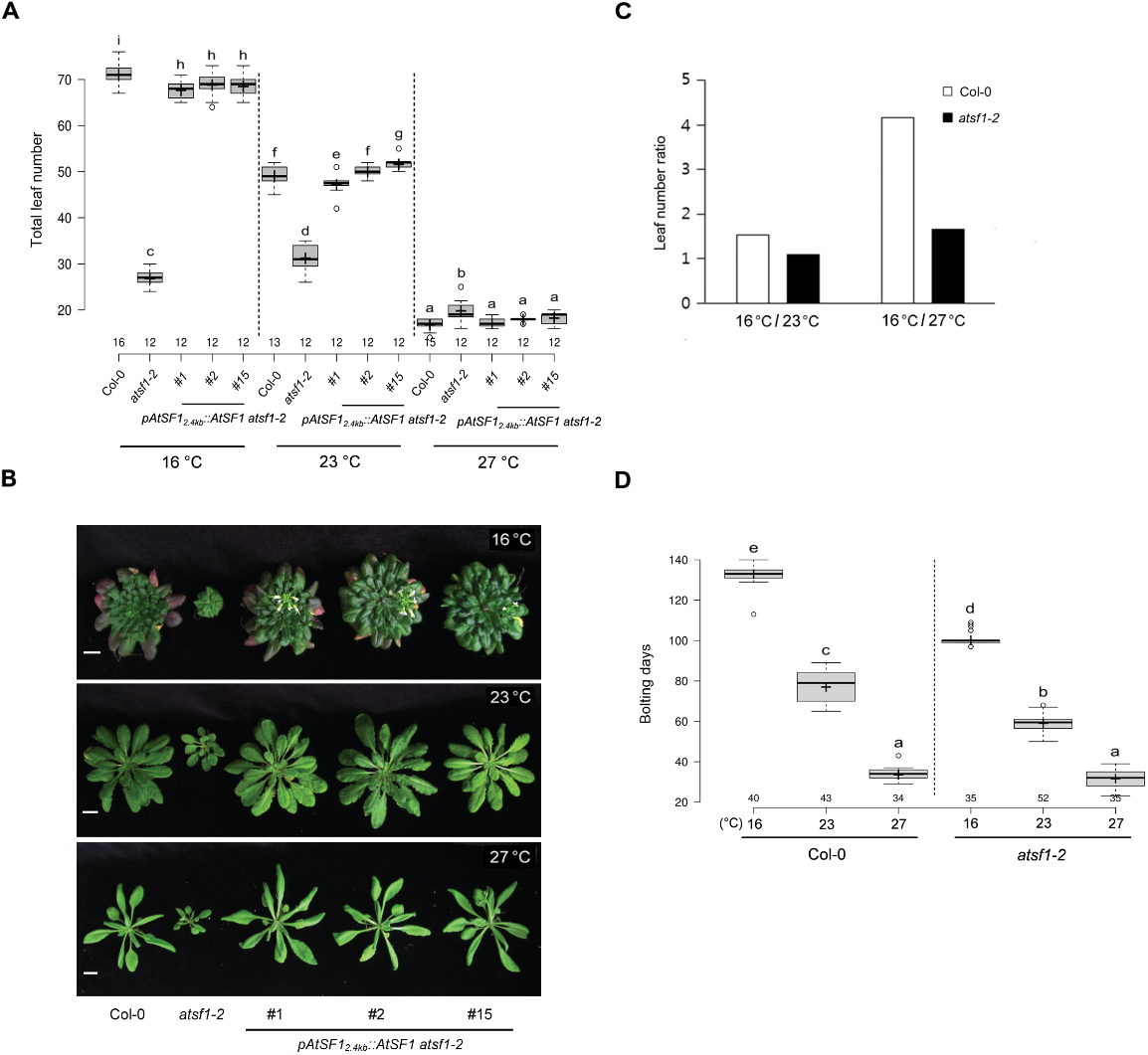


**Figure S1.** Flowering time phenotypes of *atsf1-2* mutants at different ambient temperatures under SD conditions. (A and B) Box plot (see METHODS for further information on box plots) showing flowering time (A) and phenotypes (B) of wild-type plants (Col-0), *atsf1-2* mutants, and rescued lines (*_P_AtSF1_2.4kb_::AtSF1* *atsf1-2*) grown at 27 °C, 23 °C, or 16 °C. Error bars indicate the standard error of the mean of three biological replicates. Photographs were taken when *atsf1-2* mutants flowered. Scale bars, 1 cm. (C) Leaf number ratio (LNR, 16 °C/23 °C and 16 °C/27 °C) of wild-type plants and *atsf1-2* mutants (see METHODS for further information about LNR). (D) Box plot showing bolting days of wild-type and *atsf1-2* mutants grown at 27 °C, 23 °C, and 16 °C. In (A) and (D), letters indicate statistical groups determined with multiple comparisons with the Duncan method. Multiple comparisons were performed within temperatures and within genotypes. Groups were considered statistically different when *P* < 0.05.


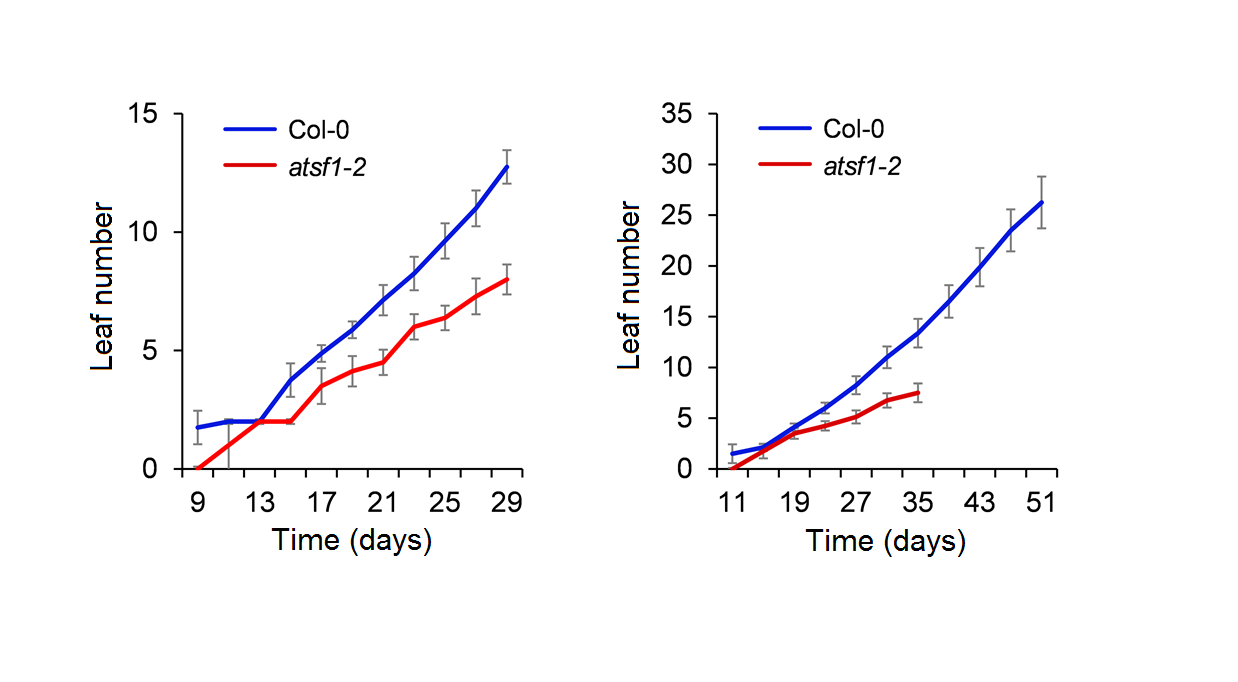


**Figure S2.** Leaf number phenotypes of wild-type (Col-0) plants and *atsf1-2* mutants at 23 °C and 16 °C under LD conditions in consecutive days after germination. Error bars indicate the standard error.


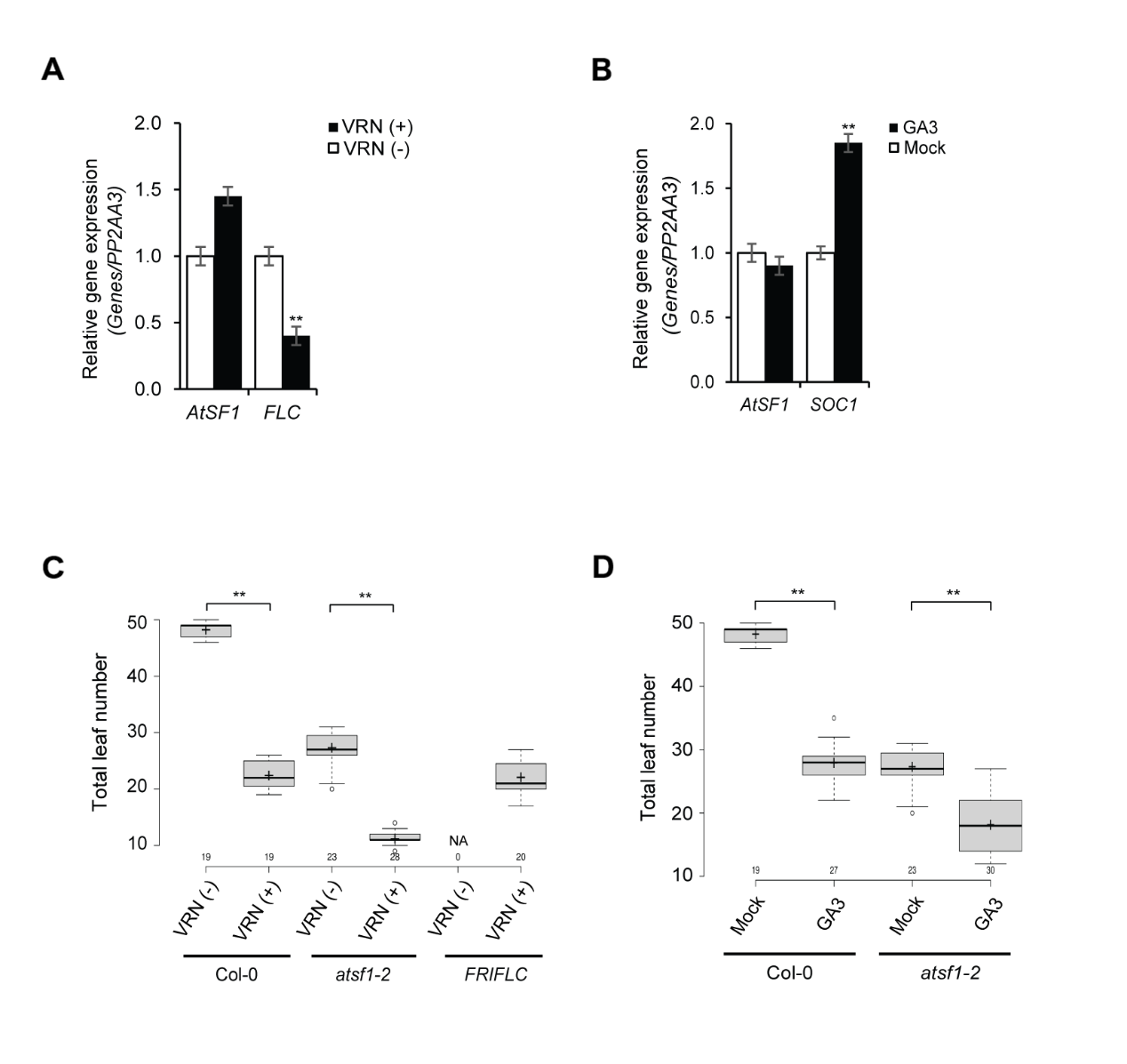


**Figure S3.** Expression of *AtSF1* and flowering time of *atsf1-2* mutants under floral inductive conditions. (A) Effects of vernalization (VRN) on *AtSF1* expression in wild-type (Col-0) plants. *AtSF1* or *FLC* expression levels in non-vernalized wild-type plants were defined as 1.0. *FLC* was used as a positive control. Samples were harvested at ZT 16. Error bars indicate the standard error of the mean of three biological replicates. Asterisks indicate statistically significant differences, as determined by Student’s *t*-test (^**^*P* < 0.01). n.s., not significant. (B) Effects of gibberellic acid (GA3) on *AtSF1* expression in wild-type plants. *AtSF1* and *SOC1* expression levels in wild-type plants without GA treatment were defined as 1.0. *SOC1* was used as a positive control. Samples were harvested at ZT 16. (C and D) Box plot showing flowering time of *atsf1-2* mutants treated for 4 weeks with vernalization (C) or 100 µM GA3 (D) until the flowering at 23 °C under SD conditions. *FRIFLC* plants were used as mock group. NA indicates that plants failed to flower during the course of the experiment.


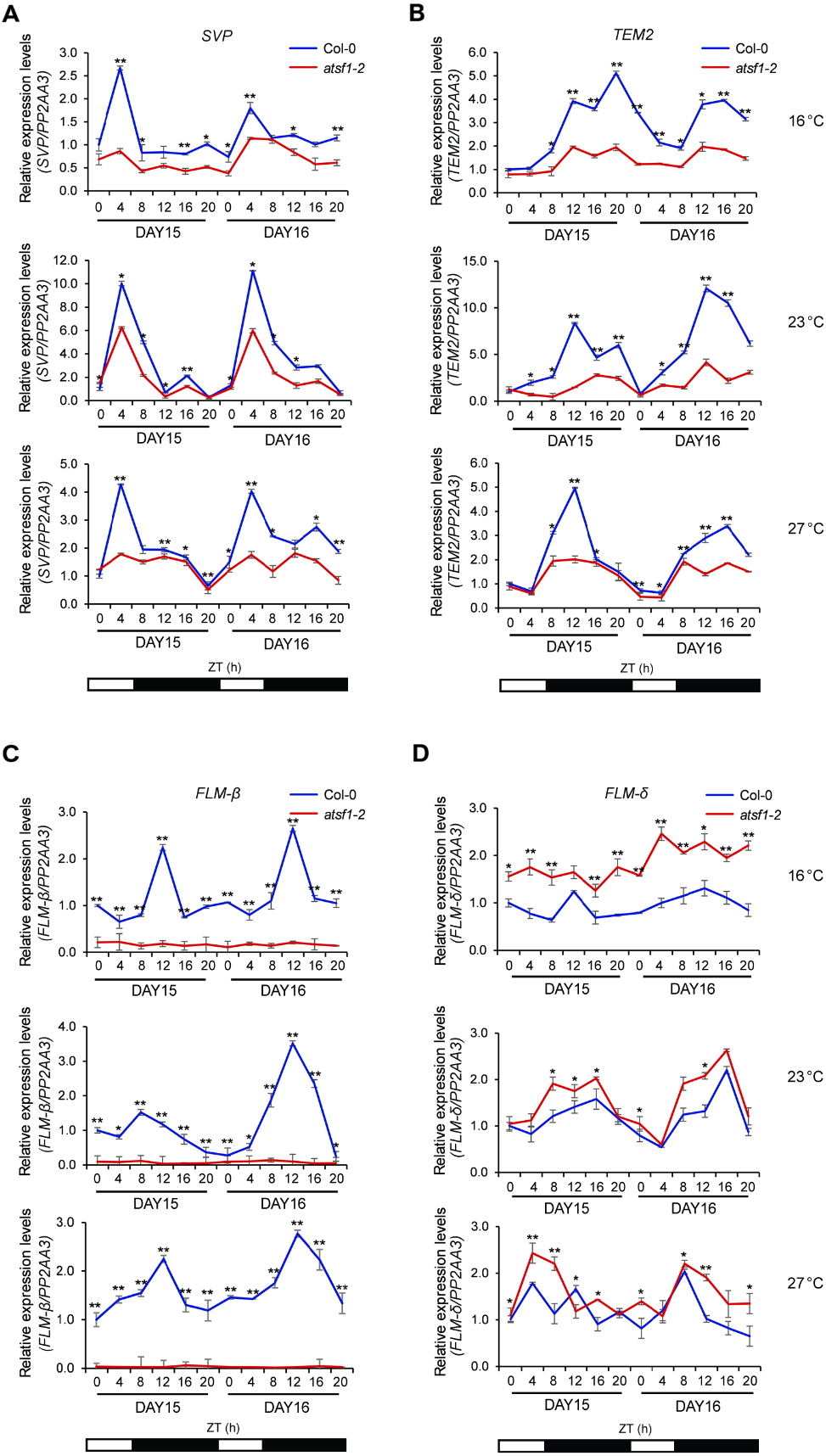


**Figure S4.** Effect of temperature on the diurnal expression of *SVP, TEM2, FLM-β,* and *FLM-δ* in the *atsf1-2* mutants under SD conditions. Expression levels of *SVP* (A)*, TEM2* (B)*, FLM-β* (C)*,* and *FLM-δ* (D) were measured by RT–qPCR (Student’s *t*-test, ^*^*P*<0.05; ^**^*P*<0.01). Expression levels in wild-type (Col-0) plants at ZT 0 on day 15 at the indicated temperatures were defined as 1.0. Error bars indicate the standard error of the mean of three biological replicates. The *PP2AA3* (*AT1G13320*) gene was used as an internal control.


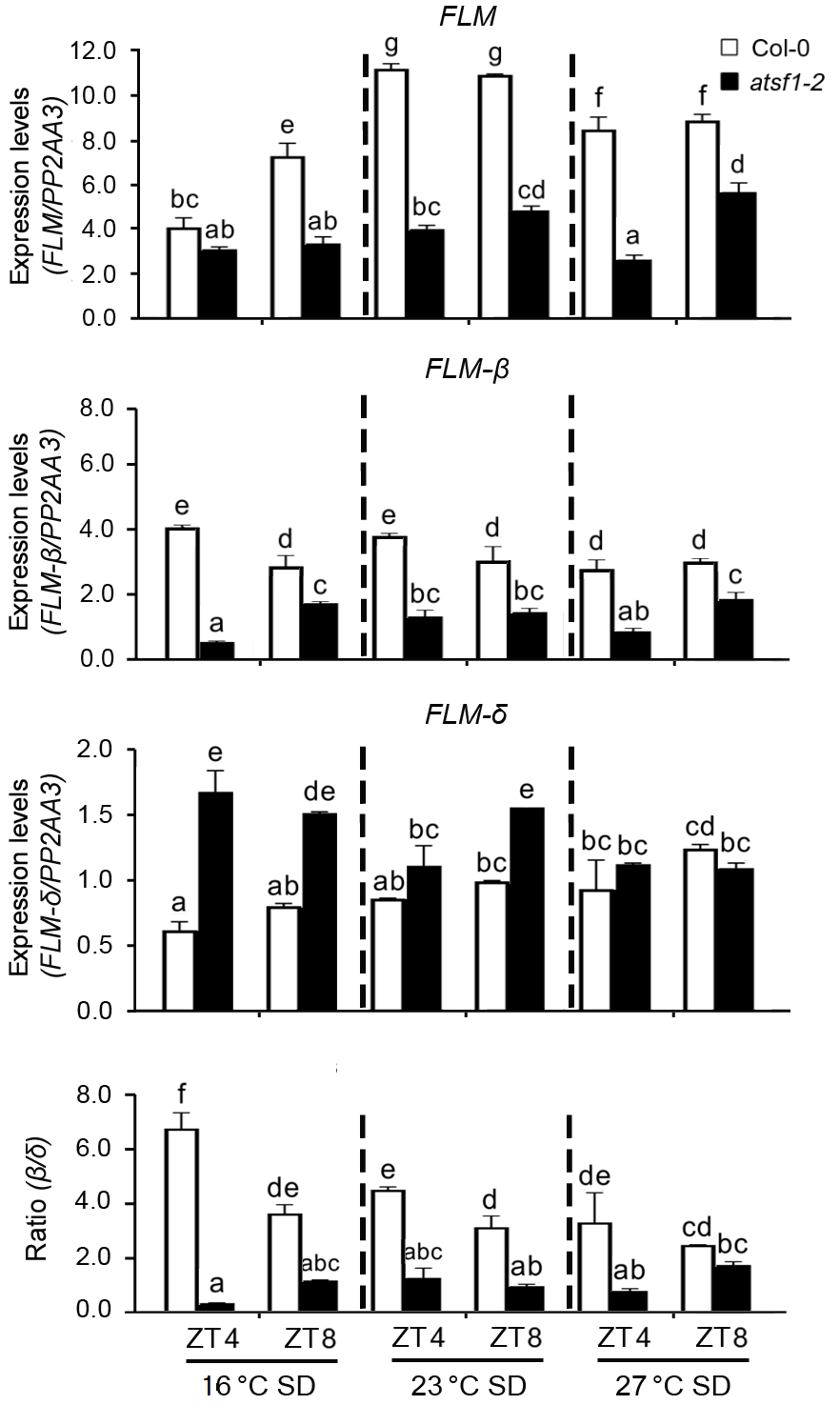


**Figure S5.** Effect of *AtSF1* mutation on AS patterns of *FLM* under SD conditions. Expression levels of *FLM*, *FLM-β,* and *FLM-δ* transcripts in 16-day-old seedlings, measured by RT–qPCR, at the indicated temperatures and time points (ZT 4 and 8). The *FLM-β*/*FLM-δ* ratio is shown below. Error bars indicate the standard error of the mean of three biological replicates. Statistical analysis was performed as described in Figure S1.


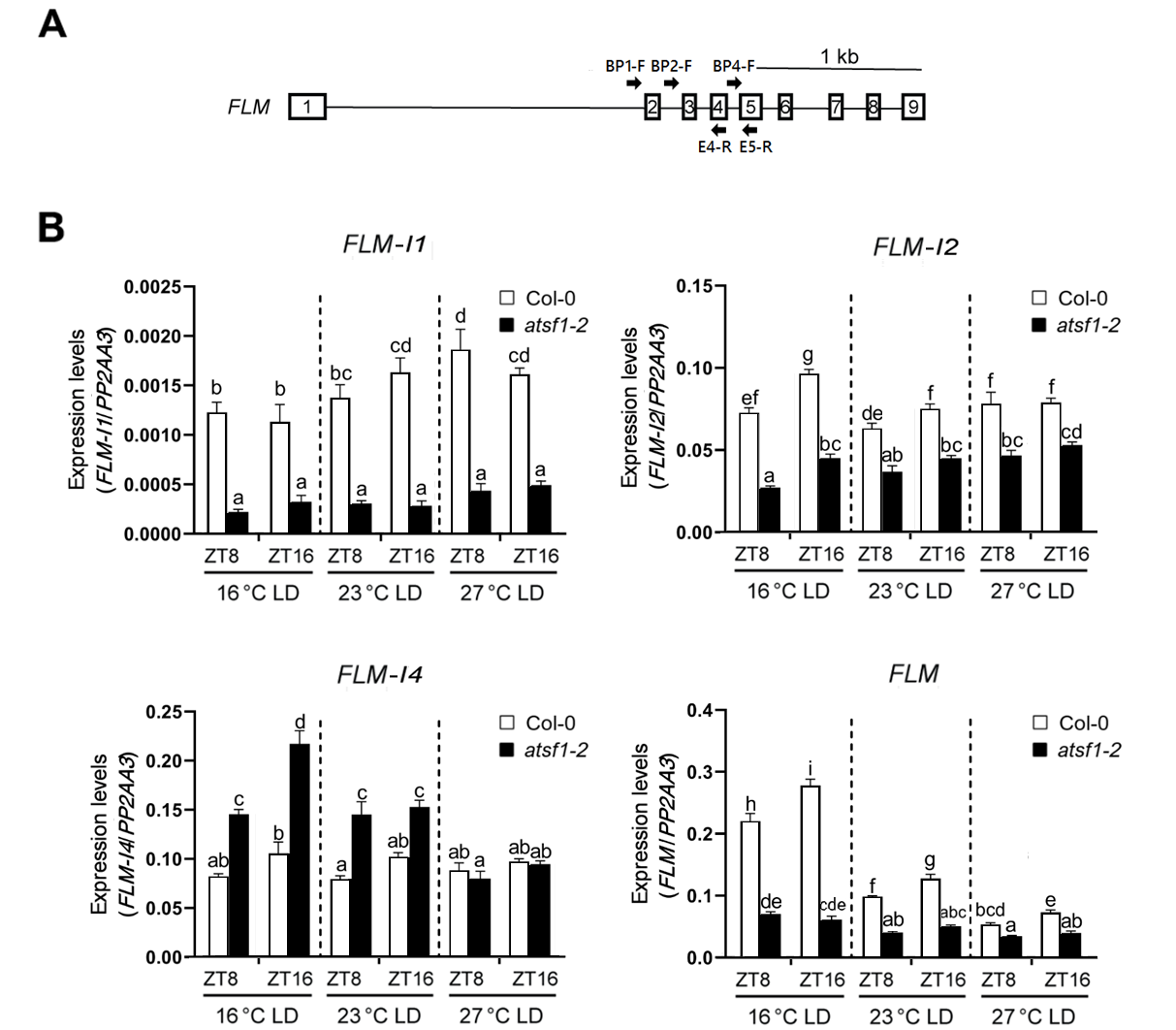


**Figure S6.** Alternative splicing patterns of *FLM* in *atsf1-2* mutants. (A) Schematic representation of the *FLM* locus. Square boxes and lines represent exons and introns, respectively. Arrows indicate primers used for RT–qPCR analysis. BP1-F and E4-R primer sets and BP2-F and E4-R primer sets amplify intron 1-specific transcripts and intron 2-specific transcripts, respectively. BP4-F and E5-R primer sets amplify intron 4-exon 5 transcript. (B) The expression of *FLM* introns 1, 2 and 4 retention forms, and *FLM* in wild-type (Col-0) plants and *atsf1-2* mutants at different temperatures. In 8-day-old (27 °C and 23 °C) or 12-day-old (16 °C) seedlings under LD conditions, RT–qPCR was performed at the indicated temperatures and time points (ZT 8 and 16). Expression levels were normalized to *PP2AA3* gene. Error bars indicate the standard error of the mean of three biological replicates. In (B), statistical analysis was performed as described in Figure S1.


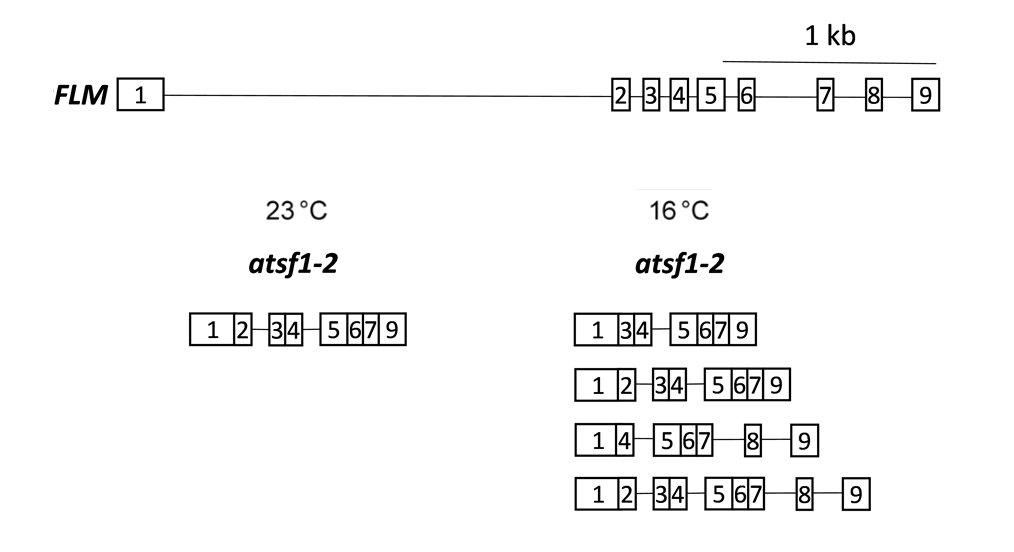


**Figure S7.** Detection of *FLM* intron retention forms in *atsf1-2* mutants. In 8-day-old (23 °C) or 12-day-old (16 °C) seedlings under LD conditions, RT–PCR was performed at the indicated temperatures and time points (ZT 16). Exons and introns indicated by boxes and lines, respectively.

**
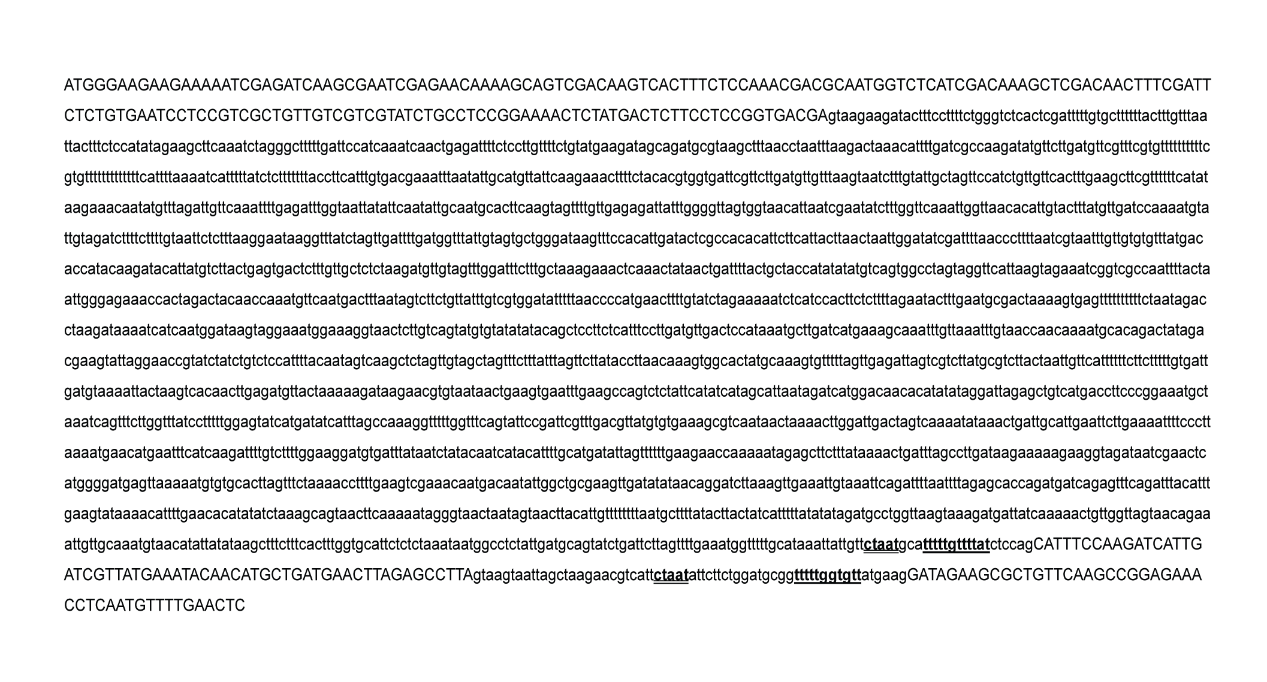
**

**Figure S8.** *In silico* prediction of BPS in introns 1 and 2 of *FLM* pre-mRNA. The putative BPS of *FLM* pre-mRNA were predicted using the online tool at <http://www.cbs.dtu.dk/services/NetPGene/>, with the following criteria: (1) consensus branch point sequence: CURAY or YURAY, where the underlined U and A are essential nucleotides; (2) located between 20 and 60 bases upstream of the 3′ splice site; (3) the branch point and the associated U-rich element are over 50 nucleotides (nts) away from the downstream 5′ splice site; (4) the associated U-rich element lying downstream of the BPS is found just upstream of the 3′ splice junction. The putative BPS is in bold and double underlined, and the putative polypyrimidine tract (Py tract) is in bold and single underlined. Capital and lowercase letters represent exons and introns, respectively.


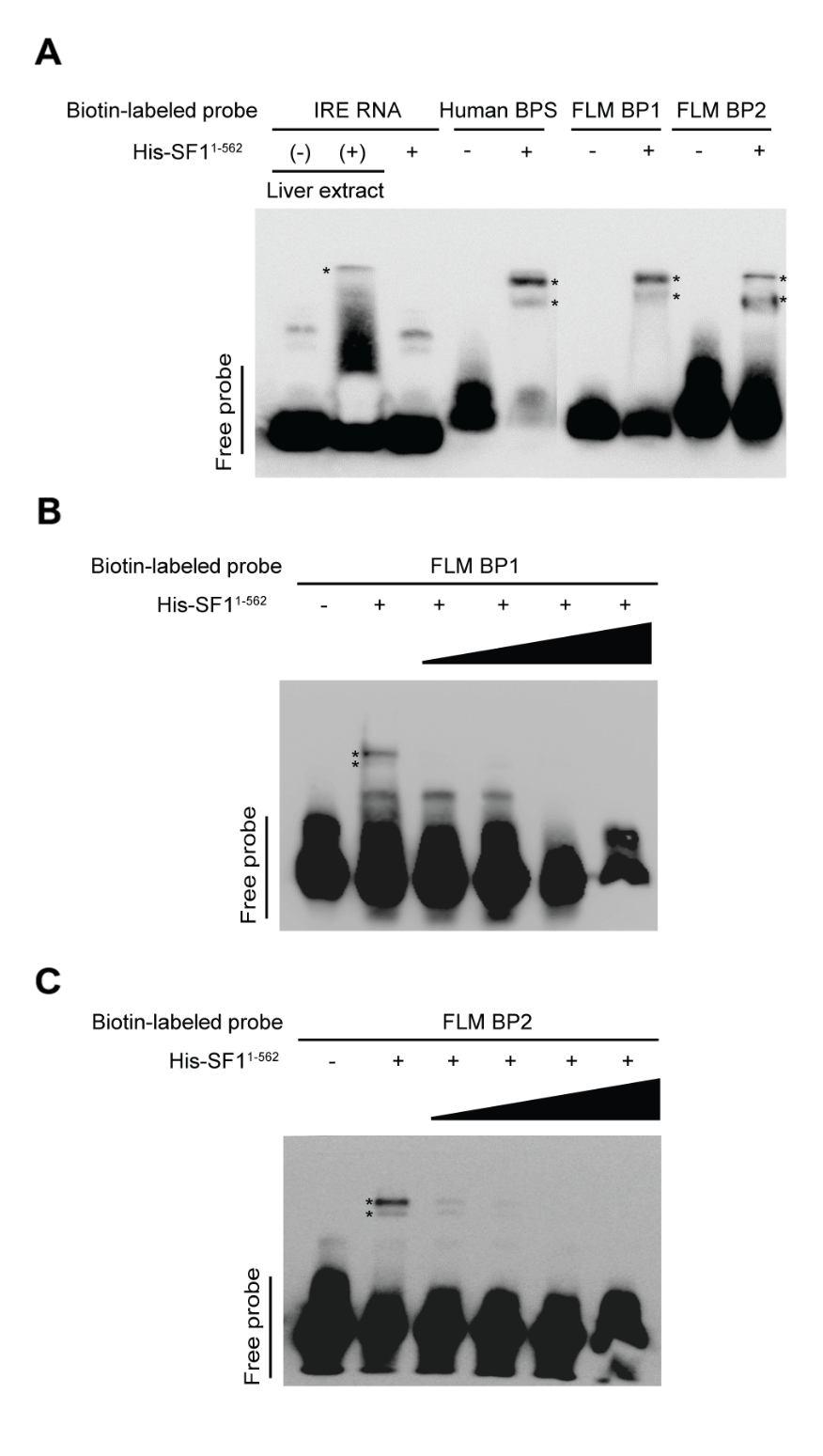


**Figure S9.** AtSF1 binds to the putative BPS sites in introns 1 and 2 of *FLM* pre-mRNA *in vitro*. (A) RNA EMSA showing the IRE RNA probe incubated with cytosolic liver extracts [(+) and (-)] provided with the RNA EMSA kit. Human RNA containing the human BPS and Py tract incubated with recombinant His-tagged truncated AtSF1 (His-SF1^1-562^) served as a positive control, whereas IRE RNA probe added to His-SF1^1-562^ served as a negative control. (B and C) Competition assay of His-SF1^1-562^ protein binding to FLM BP1 (B) and FLM BP2 (C). Unlabeled probes of identical sequences were used as competitors at 10×, 50×, 100×, 200× molar excesses. Shifted bands are indicated by asterisks and the vertical line denotes the free RNA probe.


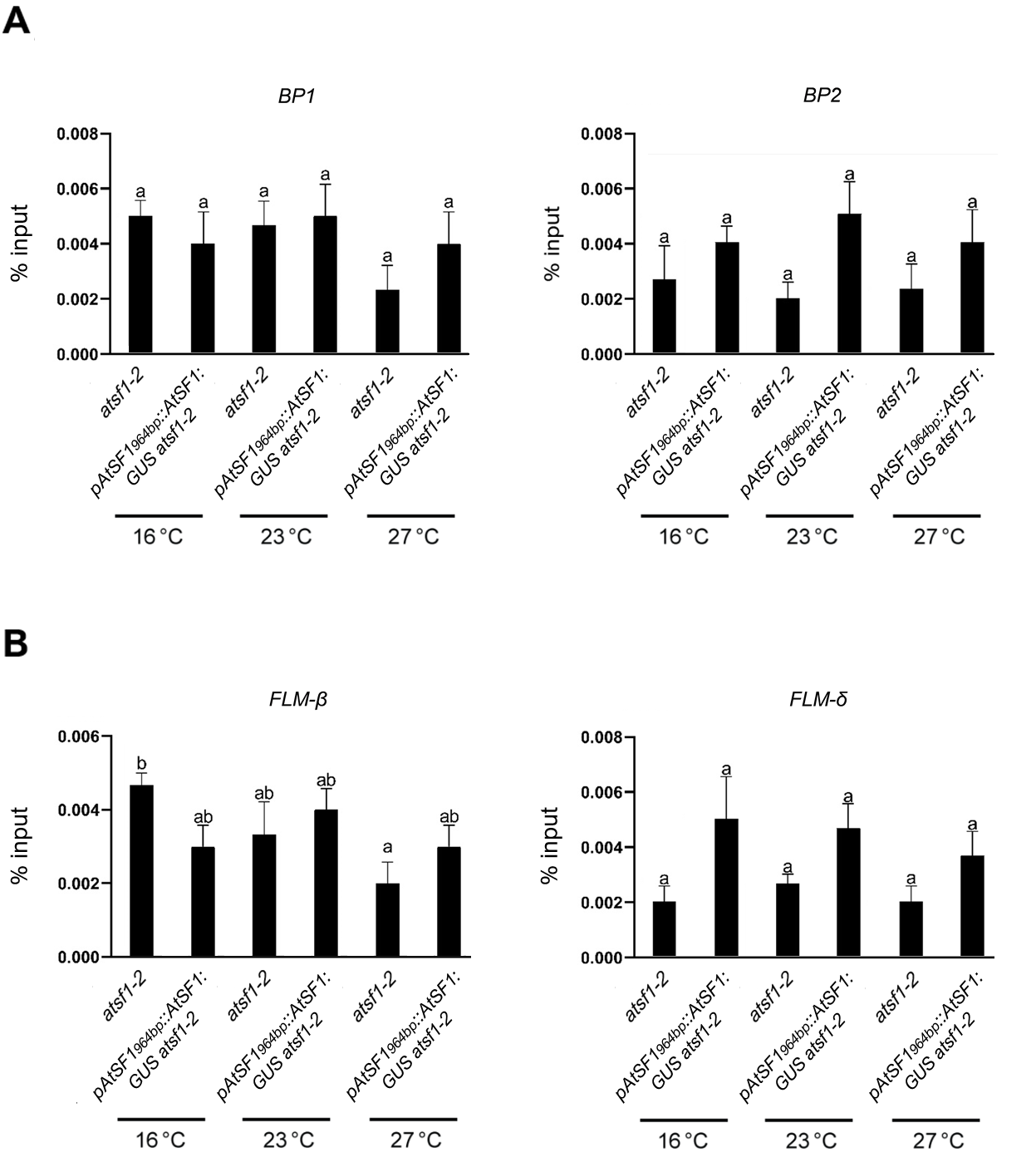


**Figure S10.** RIP analysis of AtSF1-GUS binding to *FLM* pre-mRNA using anti c-Myc antibody. 9-day-old seedlings of *pAtSF1_964bp_::AtSF1:GUS atsf1-2* and *atsf1-2* plants at different temperatures under LD conditions (ZT 16) after one day shift from 23 °C to 16 °C or 27 °C were used. The location of primers used for RIP assay was described in Figure 5. The abundance of differently spliced *FLM* transcripts was quantified by qPCR. Error bars indicate the standard error of the mean of three biological replicates. In (A) and (B), statistical analysis was performed as described in Figure S1.


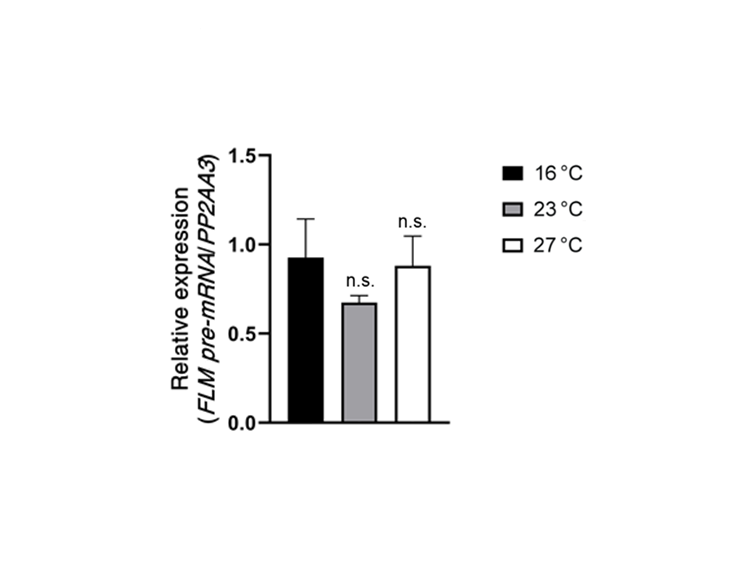


**Figure S11.** Effect of temperature shifts on the expression of *FLM* pre-mRNA. Expression levels were measured by RT-qPCR (Student’s *t*-test, n.s., not significant) at the indicated temperatures. Error bars indicate the standard error of the mean of three biological replicates.

**Table S1** Oligonucleotides used in this study.

| **Gene** | **Purpose** | **Primers (5' to 3')** | **Direction** |
| --- | --- | --- | --- |
| *FLM* | RT-qPCR | CTATGACTCTTCCTCCGGTGA | F |
|  |  | TTCAAGCTTGCTTTGGACTG | R |
| *FLM-BP4-F* | RT-qPCR | CGACACATTTTTCTCCCCTCT | F |
| *FLM-E4-R* |  | CGGACAGAGCAGTCTCAAGTT | R |
| *FLM-E5-R* |  | TCCTTGTGTGGAAGATAATTCTG | R |
| *FLM*  *pre-mRNA* | RT-qPCR | GCTTTTTTCACTTTGGTGCAT | F |
|  |  | GCCTAGAATATGGCCTTTATCGA | R |
| *FLM-β* | RT-qPCR | CATGCTGATGAACTTAGAGCCT | F |
|  |  | CAGCAACGTATTCTTTCCCAT | R |
| *FLM-δ* | RT-qPCR | GATAGAAGCGCTGTTCAAGC | F |
|  |  | CAGCAACGTATTCTTTCCCAT | R |
| *FLC* | RT-qPCR | GCCAAGAAGACCGAACTCAT | F |
|  |  | TTTGTCCAGCAGGTGACATC | R |
| *SVP*  (*At2g22540.1*) | RT-qPCR | CAAGGACTTGACATTGAAGAGCTTCA | F |
|  |  | CTGATCTCACTCATAATCTTGTCAC | R |
| *SVP2*  (*At2g22540.2*) | RT-qPCR | CCGGAAAACTGTTCGACATGAAGGAA | F |
|  |  | CTTGTCACTCTTTGTTTCAATCACAC | R |
| *TEM2* | RT-qPCR | GACTAGAGCGGCAGTTATATATTGAT | F |
|  |  | CTTTCCACCGCAAACGGCCA | R |
| *FCA-γ* | RT-qPCR | CTGGCCAGCATATAACACCA | F |
|  |  | TTGCTTTCACCCGTTAGACC | R |
| *FCA-β* | RT-qPCR | GAAGAAGAAATCCGTCCCTATTTCG | F |
|  |  | GCGAAATCTATTTGCCCAAC | R |
| *AtSF1* | RT-qPCR | CAGCAAAGCTTCCCACCAGG | F |
|  |  | GCCGGTGCAGGAGGTGTTG | R |
| *PP2AA3* | RT-qPCR | GCGGTTGTGGAGAACATGATACG | F |
|  |  | GAACCAAACACAATTCGTTGCTG | R |
| *FLM-β-F* | RIP | CATTTCCAAGATCATTGATCGT | F |
| *FLM-δ-F* |  | GATAGAAGCGCTGTTCAAGC | F |
| *FLM-β/δ*-R |  | CGACATTTGGTTCTTCAAGC | R |
| *FLM-BP1-F* | RIP | GTTCTAATGCATTTTTGTTTTATCT | F |
| *FLM-BP2-F* |  | CGTCATTCTAATATTCTTCTGGATG | F |
| *FLM-BP1/2-R* |  | TACTAAGGCTCTAAGTTCATCAGC | R |

**Table S2** Flowering time of mutants and transgenic plants used in this study.

| **Genotype** | **RLN** | **CLN** | **TLN** | **TLN SD** | **TLN range** | ***n*** |
| --- | --- | --- | --- | --- | --- | --- |
| **Experiment 1 (27 °C, long days)** | |  |  |  |  |  |
| Col-0 | 9.6 | 2.9 | 12.9 | ±0.5 | 12–13 | 21 |
| *atsf1-2* | 7.6 | 1.8 | 9.4 | ±0.7 | 9–10 | 23 |
| *pAtSF1_2.4kb_::AtSF1 atsf1-2* (#1) | 11.4 | 2.2 | 13.7 | ±0.5 | 13–14 | 21 |
| *pAtSF1_2.4kb_::AtSF1 atsf1-2* (#2) | 10.5 | 2.7 | 13.2 | ±0.8 | 12–14 | 22 |
| *pAtSF1_2.4kb_::AtSF1 atsf1-2* (#15) | 11.0 | 2.4 | 13.4 | ±0.7 | 13–14 | 21 |
| **Experiment 2 (27 °C, long days)** | |  |  |  |  |  |
| Col-0 | 8.6 | 2.9 | 11.6 | ±0.9 | 11–13 | 19 |
| *atsf1-2* | 6.4 | 2.1 | 8.5 | ±0.5 | 8–9 | 21 |
| *pAtSF1_2.4kb_::AtSF1 atsf1-2* (#1) | 9.7 | 2.0 | 11.7 | ±0.5 | 11–12 | 20 |
| *pAtSF1_2.4kb_::AtSF1 atsf1-2* (#2) | 9.9 | 2.0 | 11.9 | ±1.2 | 11–13 | 19 |
| *pAtSF1_2.4kb_::AtSF1 atsf1-2* (#15) | 9.0 | 2.1 | 11.1 | ±1.0 | 11–12 | 22 |
| **Experiment 3 (27 °C, long days)** | |  |  |  |  |  |
| Col-0 | 9.2 | 2.0 | 11.2 | ±1.0 | 10–12 | 24 |
| *atsf1-2* | 6.2 | 2.2 | 8.4 | ±0.5 | 8–9 | 19 |
| *pAtSF1_2.4kb_::AtSF1 atsf1-2* (#1) | 9.8 | 2.0 | 11.8 | ±0.9 | 11–13 | 20 |
| *pAtSF1_2.4kb_::AtSF1 atsf1-2* (#2) | 9.8 | 2.0 | 11.8 | ±1.4 | 10–13 | 24 |
| *pAtSF1_2.4kb_::AtSF1 atsf1-2* (#15) | 9.4 | 1.9 | 11.3 | ±1.0 | 10–12 | 18 |
| **Experiment 4 (23 °C, long days)** | |  |  |  |  |  |
| Col-0 | 10.9 | 2.1 | 13.1 | ±1.4 | 12–15 | 28 |
| *atsf1-2* | 7.1 | 1.9 | 8.9 | ±1.3 | 8–10 | 24 |
| *pAtSF1_2.4kb_::AtSF1 atsf1-2* (#1) | 12.6 | 2.3 | 14.8 | ±1.3 | 14–16 | 24 |
| *pAtSF1_2.4kb_::AtSF1 atsf1-2* (#2) | 12.4 | 2.1 | 14.6 | ±1.2 | 13–16 | 21 |
| *pAtSF1_2.4kb_::AtSF1 atsf1-2* (#15) | 13.2 | 2.3 | 15.4 | ±1.4 | 14–17 | 23 |
| **Experiment 5 (23 °C, long days)** | |  |  |  |  |  |
| Col-0 | 12.4 | 3.0 | 15.4 | ±0.5 | 15–16 | 21 |
| *atsf1-2* | 9.3 | 1.7 | 11.0 | ±0.6 | 10–12 | 22 |
| *pAtSF1_2.4kb_::AtSF1 atsf1-2* (#1) | 13.9 | 2.6 | 16.4 | ±1.1 | 15–18 | 21 |
| *pAtSF1_2.4kb_::AtSF1 atsf1-2* (#2) | 13.7 | 2.6 | 16.3 | ±1.1 | 15–18 | 20 |
| *pAtSF1_2.4kb_::AtSF1 atsf1-2* (#15) | 13.4 | 2.8 | 16.1 | ±1.1 | 15–17 | 24 |
| **Experiment 6 (23 °C, long days)** | |  |  |  |  |  |
| Col-0 | 13.2 | 2.6 | 15.8 | ±0.8 | 15–17 | 24 |
| *atsf1-2* | 7.6 | 2.1 | 9.7 | ±0.5 | 9–10 | 22 |
| *pAtSF1_2.4kb_::AtSF1 atsf1-2* (#1) | 13.1 | 2.7 | 15.8 | ±1.3 | 15–17 | 24 |
| *pAtSF1_2.4kb_::AtSF1 atsf1-2* (#2) | 13.8 | 2.9 | 16.7 | ±0.9 | 16–18 | 24 |
| *pAtSF1_2.4kb_::AtSF1 atsf1-2* (#15) | 13.1 | 2.9 | 15.9 | ±0.9 | 15–17 | 22 |
| **Experiment 7 (16 °C, long days)** | |  |  |  |  |  |
| Col-0 | 24.1 | 4.9 | 29.0 | ±0.9 | 28–30 | 25 |
| *atsf1-2* | 7.5 | 2.0 | 9.5 | ±1.0 | 9–11 | 20 |
| *pAtSF1_2.4kb_::AtSF1 atsf1-2* (#1) | 26.1 | 4.4 | 30.5 | ±1.2 | 29–32 | 18 |
| *pAtSF1_2.4kb_::AtSF1 atsf1-2* (#2) | 26.5 | 4.6 | 31.1 | ±1.2 | 30–32 | 19 |
| *pAtSF1_2.4kb_::AtSF1 atsf1-2* (#15) | 26.4 | 4.5 | 30.9 | ±1.0 | 30–32 | 19 |
| **Experiment 8 (16 °C, long days)** | |  |  |  |  |  |
| Col-0 | 24.8 | 4.4 | 29.2 | ±1.1 | 28–30 | 20 |
| *atsf1-2* | 6.9 | 1.9 | 8.8 | ±0.9 | 8–10 | 23 |
| *pAtSF1_2.4kb_::AtSF1 atsf1-2* (#1) | 25.9 | 4.3 | 30.2 | ±1.8 | 28–32 | 21 |
| *pAtSF1_2.4kb_::AtSF1 atsf1-2* (#2) | 25.1 | 3.8 | 28.9 | ±1.2 | 28–30 | 24 |
| *pAtSF1_2.4kb_::AtSF1 atsf1-2* (#15) | 22.3 | 4.1 | 26.5 | ±1.4 | 25–28 | 22 |
| **Experiment 9 (16 °C, long days)** | |  |  |  |  |  |
| Col-0 | 27.7 | 5.0 | 32.7 | ±1.6 | 31–34 | 24 |
| *atsf1-2* | 8.6 | 2.0 | 10.6 | ±0.9 | 10–12 | 26 |
| *pAtSF1_2.4kb_::AtSF1 atsf1-2* (#1) | 29.0 | 5.1 | 34.1 | ±2.9 | 31–37 | 21 |
| *pAtSF1_2.4kb_::AtSF1 atsf1-2* (#2) | 29.7 | 5.0 | 34.7 | ±1.8 | 33–37 | 19 |
| *pAtSF1_2.4kb_::AtSF1 atsf1-2* (#15) | 29.6 | 5.0 | 34.6 | ±2.3 | 32–37 | 22 |
| **Experiment 10 (27 °C, short days)** | |  |  |  |  |  |
| Col-0 | 14.3 | 2.7 | 16.9 | ±1.2 | 16–18 | 28 |
| *atsf1-2* | 17.0 | 2.7 | 19.7 | ±2.3 | 17–22 | 23 |
| *pAtSF1_2.4kb_::AtSF1 atsf1-2* (#1) | 14.8 | 2.4 | 17.2 | ±1.3 | 16–19 | 15 |
| *pAtSF1_2.4kb_::AtSF1 atsf1-2* (#2) | 16.0 | 2.0 | 18.0 | ±0.8 | 17–19 | 18 |
| *pAtSF1_2.4kb_::AtSF1 atsf1-2* (#15) | 16.0 | 2.3 | 18.3 | ±2.1 | 16–20 | 17 |
| **Experiment 11 (27 °C, short days)** | |  |  |  |  |  |
| Col-0 | 13.7 | 2.3 | 16.0 | ±0.9 | 15–17 | 25 |
| *atsf1-2* | 14.2 | 2.0 | 16.2 | ±0.8 | 15–17 | 22 |
| *pAtSF1_2.4kb_::AtSF1 atsf1-2* (#1) | 15.8 | 2.3 | 18.1 | ±0.8 | 17–19 | 18 |
| *pAtSF1_2.4kb_::AtSF1 atsf1-2* (#2) | 16.0 | 2.3 | 18.3 | ±1.3 | 17–20 | 20 |
| *pAtSF1_2.4kb_::AtSF1 atsf1-2* (#15) | 14.1 | 2.0 | 16.1 | ±1.1 | 15–17 | 21 |
| **Experiment 12 (27 °C, short days)** | |  |  |  |  |  |
| Col-0 | 17.4 | 2.1 | 19.5 | ±2.6 | 17–22 | 21 |
| *atsf1-2* | 15.2 | 2.0 | 17.2 | ±3.1 | 14–20 | 30 |
| *pAtSF1_2.4kb_::AtSF1 atsf1-2* (#1) | 18.1 | 2.0 | 20.1 | ±2.5 | 18–23 | 20 |
| *pAtSF1_2.4kb_::AtSF1 atsf1-2* (#2) | 18.6 | 1.8 | 20.4 | ±1.7 | 19–22 | 21 |
| *pAtSF1_2.4kb_::AtSF1 atsf1-2* (#15) | 17.1 | 2.1 | 19.3 | ±2.1 | 17–21 | 20 |
| **Experiment 13 (23 °C, short days)** | |  |  |  |  |  |
| Col-0 | 44.4 | 4.8 | 49.2 | ±2.0 | 47–51 | 24 |
| *atsf1-2* | 28.7 | 2.7 | 31.3 | ±2.9 | 28–34 | 23 |
| *pAtSF1_2.4kb_::AtSF1 atsf1-2* (#1) | 42.1 | 5.1 | 47.2 | ±2.4 | 45–50 | 18 |
| *pAtSF1_2.4kb_::AtSF1 atsf1-2* (#2) | 45.0 | 5.1 | 50.1 | ±1.4 | 49–52 | 19 |
| *pAtSF1_2.4kb_::AtSF1 atsf1-2* (#15) | 47.0 | 5.0 | 52.0 | ±1.7 | 50–54 | 15 |
| **Experiment 14 (23 °C, short days)** | |  |  |  |  |  |
| Col-0 | 41.1 | 4.3 | 45.4 | ±1.1 | 44–47 | 18 |
| *atsf1-2* | 21.7 | 3.2 | 24.9 | ±3.5 | 21–28 | 22 |
| *pAtSF1_2.4kb_::AtSF1 atsf1-2* (#1) | 40.1 | 3.8 | 43.9 | ±2.2 | 42–46 | 20 |
| *pAtSF1_2.4kb_::AtSF1 atsf1-2* (#2) | 43.2 | 3.8 | 46.7 | ±2.4 | 44–49 | 21 |
| *pAtSF1_2.4kb_::AtSF1 atsf1-2* (#15) | 41.9 | 4.0 | 45.9 | ±2.6 | 43–49 | 22 |
| **Experiment 15 (23 °C, short days)** | |  |  |  |  |  |
| Col-0 | 44.8 | 3.0 | 47.8 | ±1.2 | 47–49 | 19 |
| *atsf1-2* | 22.0 | 2.6 | 24.6 | ±1.8 | 23–26 | 29 |
| *pAtSF1_2.4kb_::AtSF1 atsf1-2* (#1) | 45.2 | 2.8 | 48.0 | ±0.9 | 47–49 | 20 |
| *pAtSF1_2.4kb_::AtSF1 atsf1-2* (#2) | 44.2 | 3.0 | 47.2 | ±1.5 | 46–49 | 22 |
| *pAtSF1_2.4kb_::AtSF1 atsf1-2* (#15) | 45.8 | 3.6 | 49.4 | ±1.2 | 48–51 | 21 |
| **Experiment 16 (16 °C, short days)** | |  |  |  |  |  |
| Col-0 | 64.4 | 6.7 | 71.1 | ±2.2 | 69–73 | 20 |
| *atsf1-2* | 25.0 | 1.9 | 26.9 | ±1.7 | 25–29 | 21 |
| *pAtSF1_2.4kb_::AtSF1 atsf1-2* (#1) | 60.5 | 7.2 | 67.7 | ±1.9 | 66–70 | 20 |
| *pAtSF1_2.4kb_::AtSF1 atsf1-2* (#2) | 61.9 | 7.0 | 68.9 | ±3.0 | 66–72 | 18 |
| *pAtSF1_2.4kb_::AtSF1 atsf1-2* (#15) | 61.8 | 6.8 | 68.6 | ±2.6 | 66–71 | 18 |
| **Experiment 17 (16 °C, short days)** | |  |  |  |  |  |
| Col-0 | 69.3 | 3.9 | 73.1 | ±2.5 | 71–76 | 18 |
| *atsf1-2* | 28.4 | 3.0 | 31.4 | ±1.3 | 30–33 | 19 |
| *pAtSF1_2.4kb_::AtSF1 atsf1-2* (#1) | 65.6 | 4.6 | 70.2 | ±3.5 | 67–74 | 17 |
| *pAtSF1_2.4kb_::AtSF1 atsf1-2* (#2) | 65.8 | 4.0 | 69.8 | ±2.8 | 67–73 | 17 |
| *pAtSF1_2.4kb_::AtSF1 atsf1-2* (#15) | 64.4 | 4.2 | 68.6 | ±1.5 | 67–70 | 18 |
| **Experiment 18 (16 °C, short days)** | |  |  |  |  |  |
| Col-0 | 69.0 | 4.8 | 73.8 | ±3.5 | 70–77 | 19 |
| *atsf1-2* | 27.1 | 3.2 | 30.3 | ±2.5 | 28–33 | 21 |
| *pAtSF1_2.4kb_::AtSF1 atsf1-2* (#1) | 68.8 | 4.4 | 73.2 | ±4.7 | 69–78 | 18 |
| *pAtSF1_2.4kb_::AtSF1 atsf1-2* (#2) | 69.8 | 3.8 | 73.6 | ±2.7 | 71–76 | 17 |
| *pAtSF1_2.4kb_::AtSF1 atsf1-2* (#15) | 70.4 | 4.6 | 75.0 | ±3.2 | 72–78 | 17 |
| **Experiment 19 (23 °C, long days)** | |  |  |  |  |  |
| Col-0 | 10.0 | 1.8 | 11.8 | ±0.8 | 11–13 | 22 |
| *atsf1-2* | 6.6 | 2.2 | 8.8 | ±0.8 | 8–10 | 16 |
| *pAtSF1_2.4kb_::AtSF1 atsf1-2* (#1) | 9.6 | 1.8 | 11.3 | ±0.5 | 11–12 | 23 |
| *abh1-285* | 9.1 | 2.0 | 11.1 | ±0.8 | 10–12 | 16 |
| Col-gl1 | 8.9 | 1.4 | 10.4 | ±1.4 | 9–12 | 17 |
| *sta1-1* | 7.7 | 1.5 | 9.2 | ±1.0 | 8–10 | 18 |
| **Experiment 20 (23 °C, long days)** | |  |  |  |  |  |
| Col-0 | 10.3 | 1.8 | 12.1 | ±0.4 | 12–13 | 20 |
| *atsf1-2* | 7.0 | 2.0 | 8.9 | ±0.7 | 8–10 | 22 |
| *pAtSF1_2.4kb_::AtSF1 atsf1-2* (#1) | 11.2 | 1.7 | 12.8 | ±0.9 | 12–14 | 20 |
| *abh1-285* | 9.6 | 1.0 | 10.7 | ±0.5 | 10–11 | 23 |
| Col-gl1 | 10.6 | 1.7 | 12.3 | ±0.6 | 12–13 | 19 |
| *sta1-1* | 9.0 | 1.6 | 10.5 | ±0.6 | 10–11 | 21 |
| **Experiment 21 (23 °C, long days)** | |  |  |  |  |  |
| Col-0 | 10.6 | 2.1 | 12.6 | ±0.5 | 12–13 | 20 |
| *atsf1-2* | 6.1 | 1.9 | 8.1 | ±0.6 | 8–9 | 21 |
| *pAtSF1_2.4kb_::AtSF1 atsf1-2* (#1) | 9.3 | 1.8 | 11.1 | ±1.1 | 10–12 | 23 |
| *abh1-285* | 9.5 | 2.4 | 11.9 | ±1.0 | 11–13 | 24 |
| Col-gl1 | 10.6 | 1.6 | 12.1 | ±0.7 | 11–13 | 17 |
| *sta1-1* | 9.5 | 1.2 | 10.7 | ±0.5 | 10–11 | 21 |
| **Experiment 22 (16 °C, long days)** | |  |  |  |  |  |
| Col-0 | 21.3 | 4.1 | 25.4 | ±1.4 | 24–27 | 29 |
| *atsf1-2* | 6.6 | 1.7 | 8.4 | ±0.5 | 8–9 | 25 |
| *pAtSF1_2.4kb_::AtSF1 atsf1-2* (#1) | 25.1 | 3.8 | 28.8 | ±1.2 | 28–30 | 19 |
| *abh1-285* | 17.8 | 3.6 | 21.4 | ±1.9 | 20–23 | 23 |
| Col-gl1 | 20.5 | 2.2 | 22.7 | ±0.5 | 22–23 | 21 |
| *sta1-1* | 12.1 | 2.0 | 14.1 | ±1.0 | 13–15 | 22 |
| **Experiment 23 (16 °C, long days)** | |  |  |  |  |  |
| Col-0 | 22.2 | 4.7 | 26.7 | ±1.4 | 25–28 | 22 |
| *atsf1-2* | 7.9 | 1.8 | 9.6 | ±0.9 | 9–11 | 19 |
| *pAtSF1_2.4kb_::AtSF1 atsf1-2* (#1) | 22.0 | 3.9 | 25.9 | ±0.7 | 25–27 | 20 |
| *abh1-285* | 19.7 | 3.6 | 23.2 | ±2.1 | 21–25 | 23 |
| Col-gl1 | 20.8 | 3.7 | 24.4 | ±2.2 | 22–27 | 24 |
| *sta1-1* | 10.8 | 2.2 | 12.9 | ±1.3 | 12–14 | 18 |
| **Experiment 24 (16 °C, long days)** | |  |  |  |  |  |
| Col-0 | 19.8 | 4.3 | 24.1 | ±0.9 | 23–25 | 22 |
| *atsf1-2* | 7.8 | 1.3 | 9.1 | ±0.5 | 9–10 | 18 |
| *pAtSF1_2.4kb_::AtSF1 atsf1-2* (#1) | 20.8 | 3.3 | 24.1 | ±0.9 | 23–25 | 20 |
| *abh1-285* | 18.7 | 3.9 | 22.5 | ±1.2 | 21–24 | 21 |
| Col-gl1 | 19.8 | 3.4 | 23.2 | ±0.8 | 22–24 | 24 |
| *sta1-1* | 13.6 | 2.8 | 16.4 | ±1.2 | 15–18 | 20 |

RLN: rosette leaf number, CLN: cauline leaf number, TLN: total leaf number
