# Supplementary figures and images for: Role of Arabidopsis Splicing factor SF1 in Temperature-Responsive Alternative Splicing of FLM pre-mRNA
Source: Front Plant Sci. 2020 Dec 1;11:596354. doi: 10.3389/fpls.2020.596354 (PMC7735993; doi:10.3389/fpls.2020.596354)

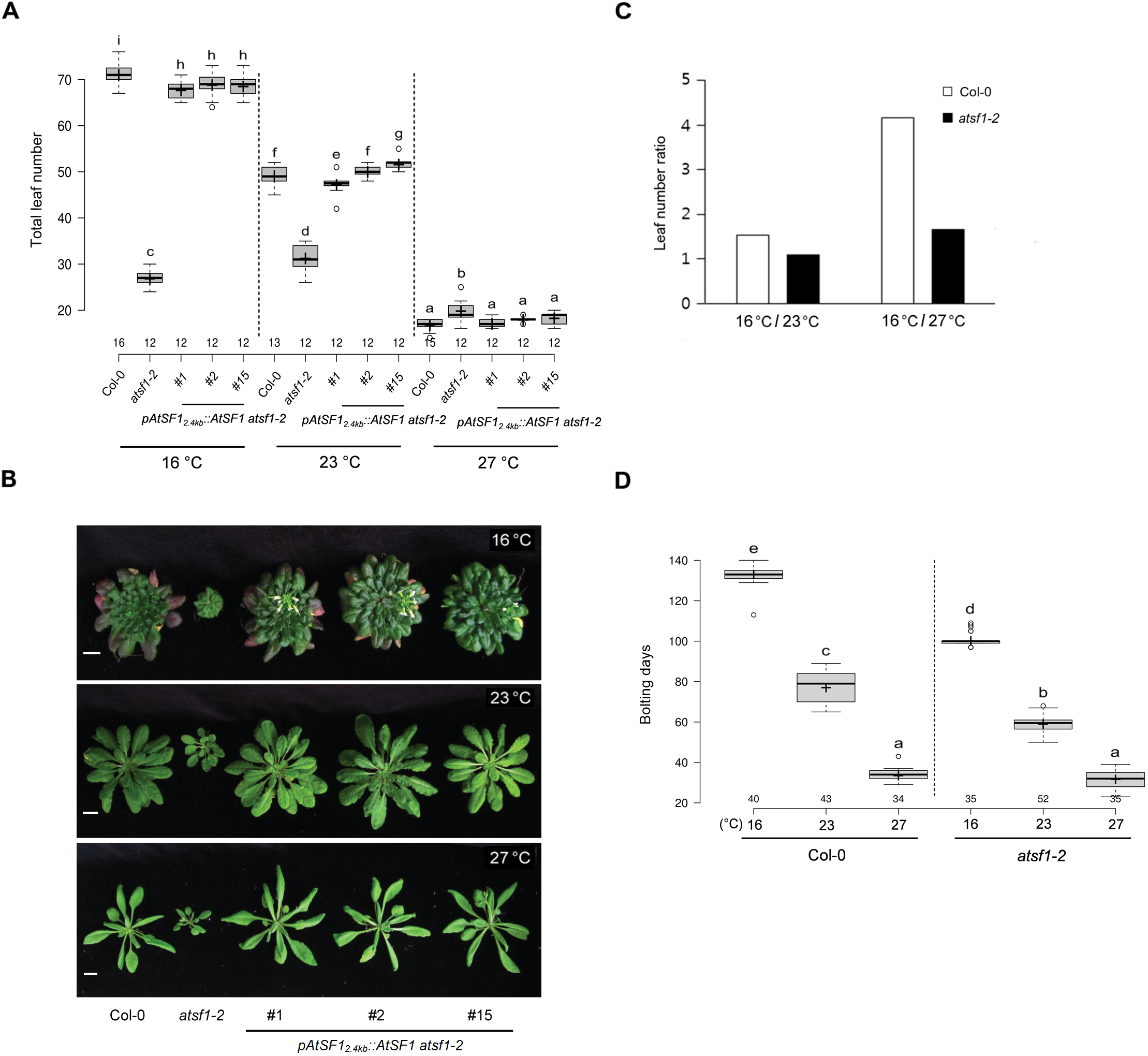

Supplement: Supplementary file 2 [file Image_1.TIF]

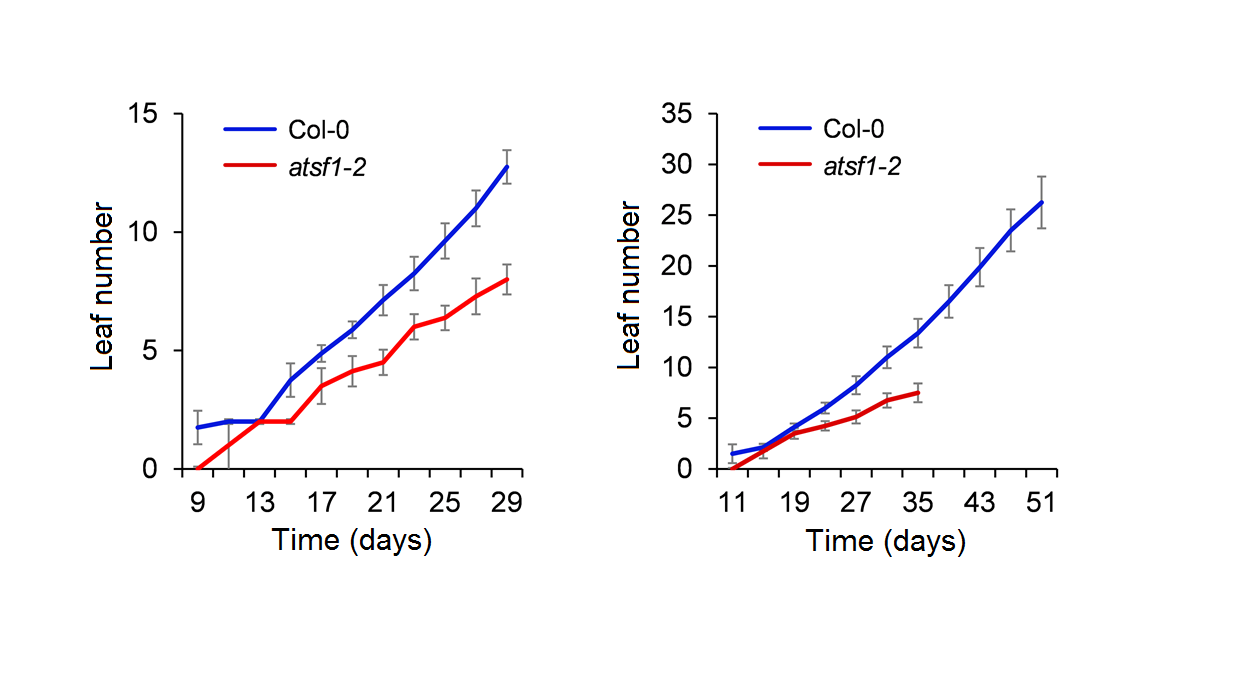

Supplement: Supplementary file 3 [file Image_2.TIF]

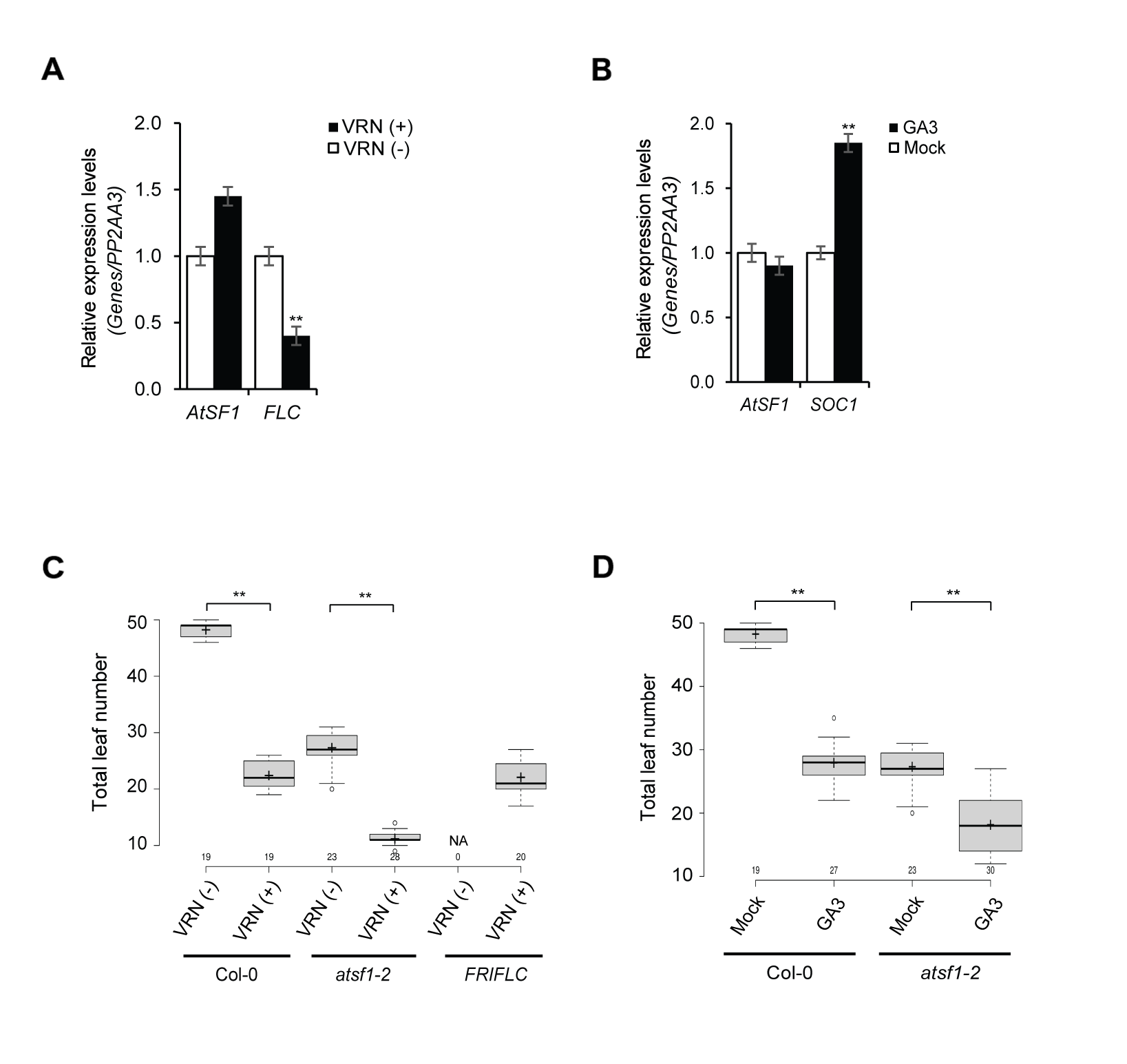

Supplement: Supplementary file 4 [file Image_3.TIF]

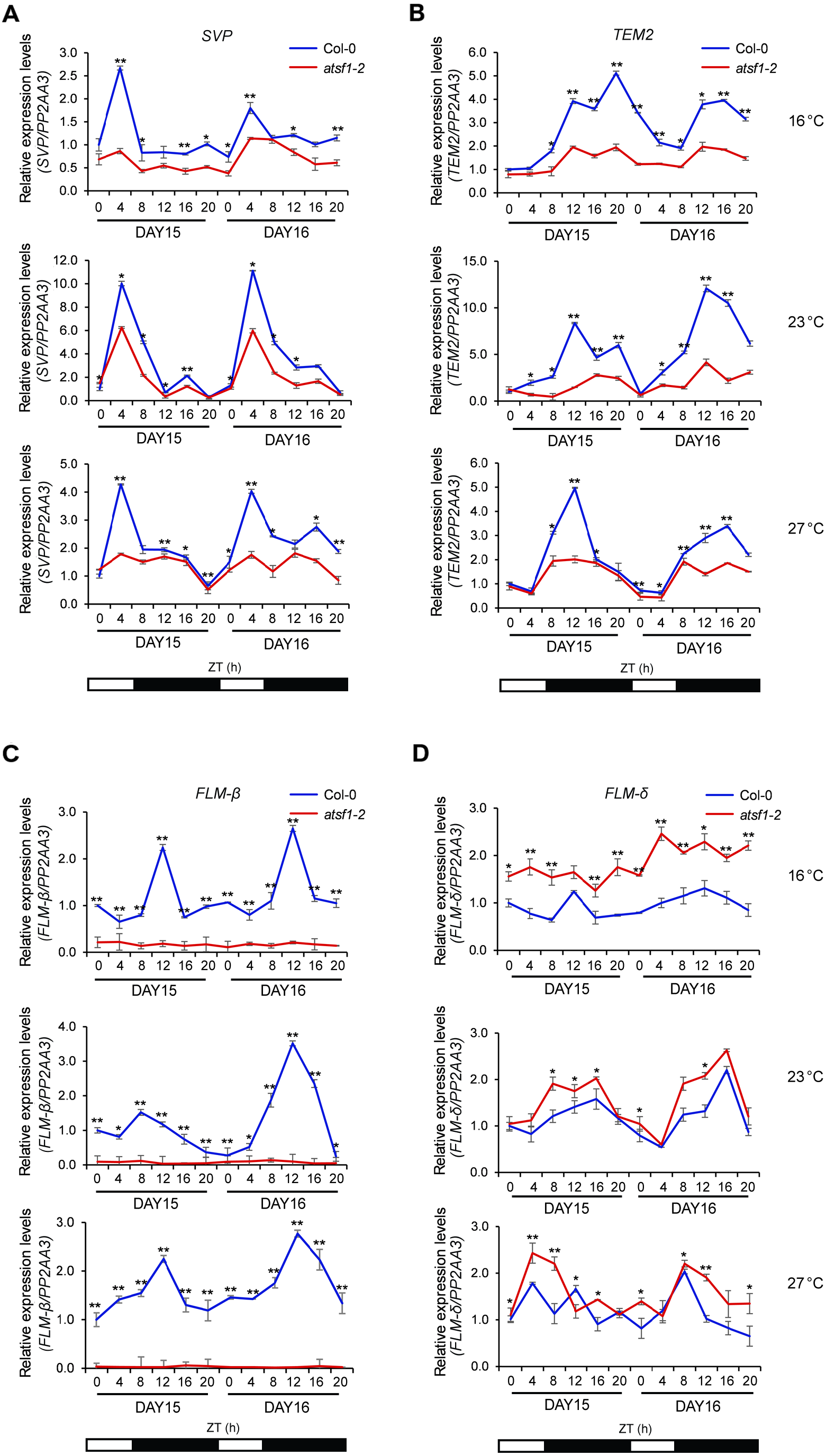

Supplement: Supplementary file 5 [file Image_4.TIF]

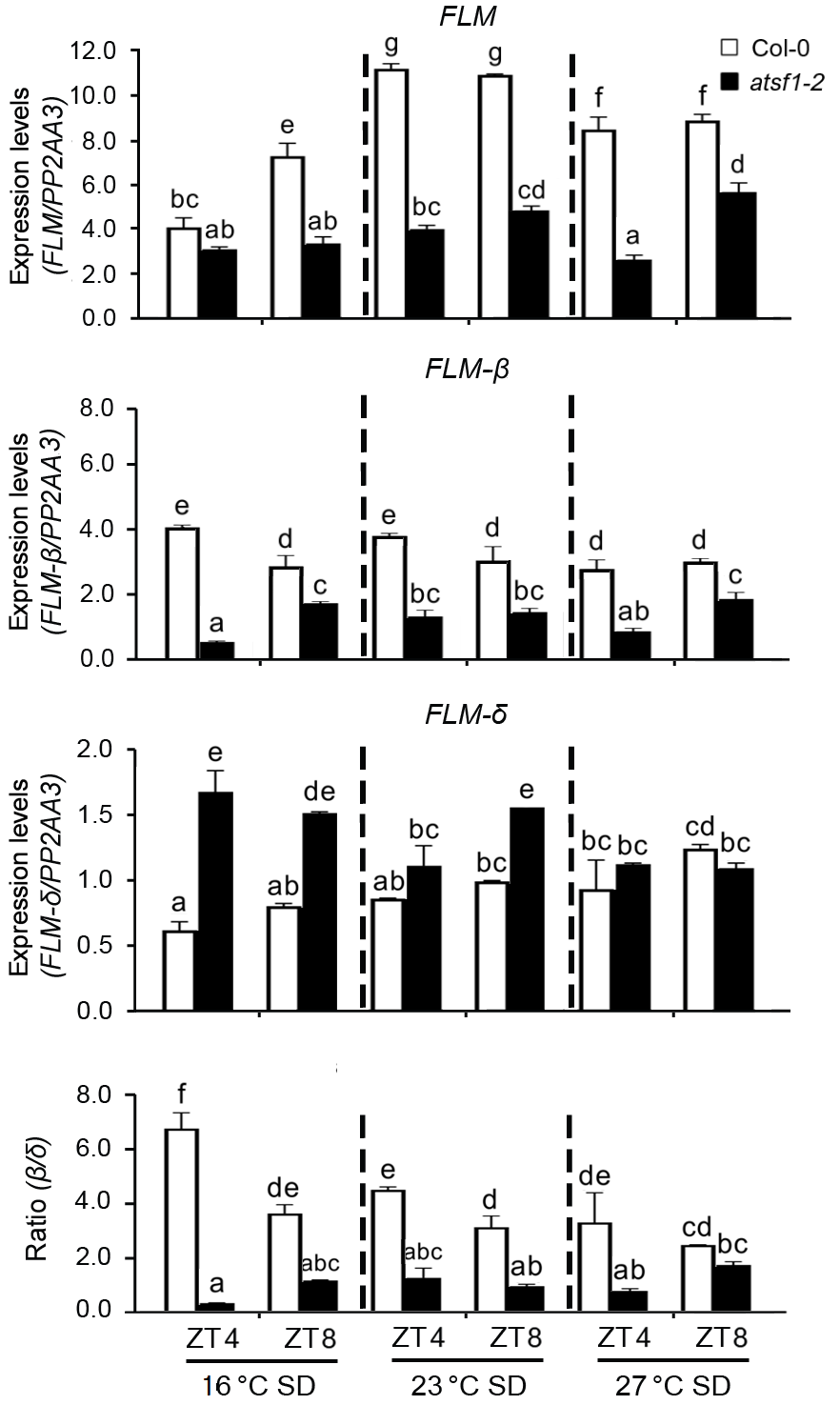

Supplement: Supplementary file 6 [file Image_5.TIF]

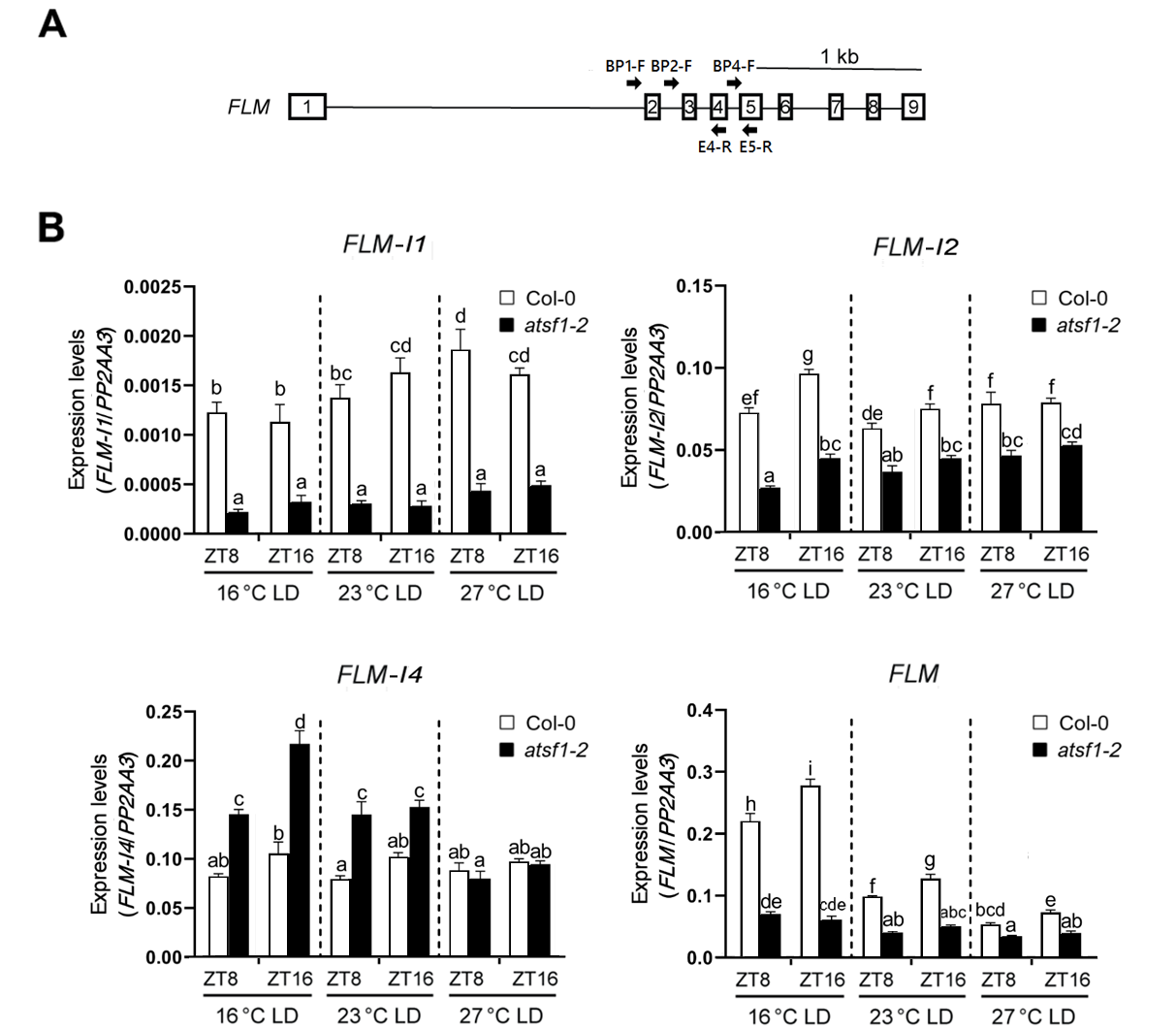

Supplement: Supplementary file 7 [file Image_6.TIF]

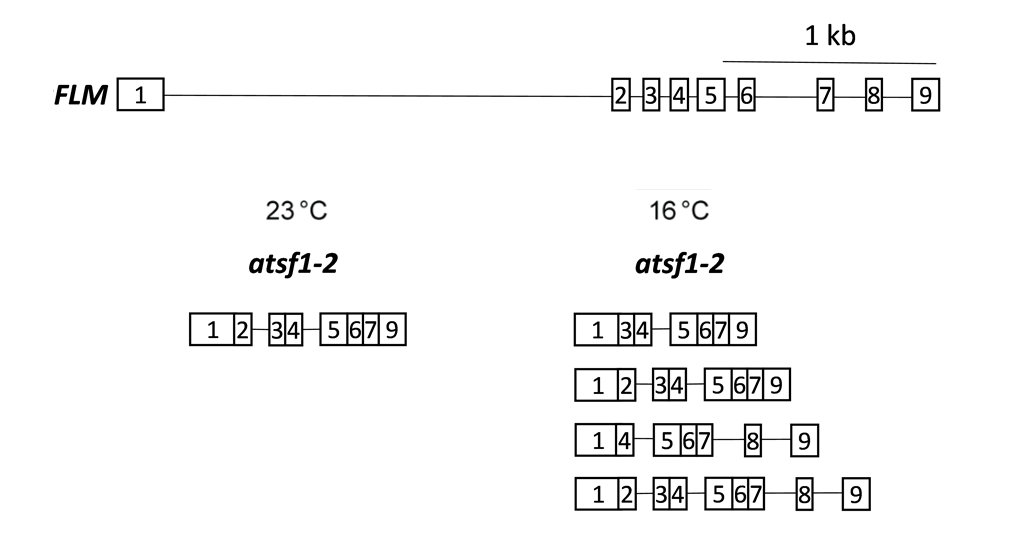

Supplement: Supplementary file 8 [file Image_7.TIF]

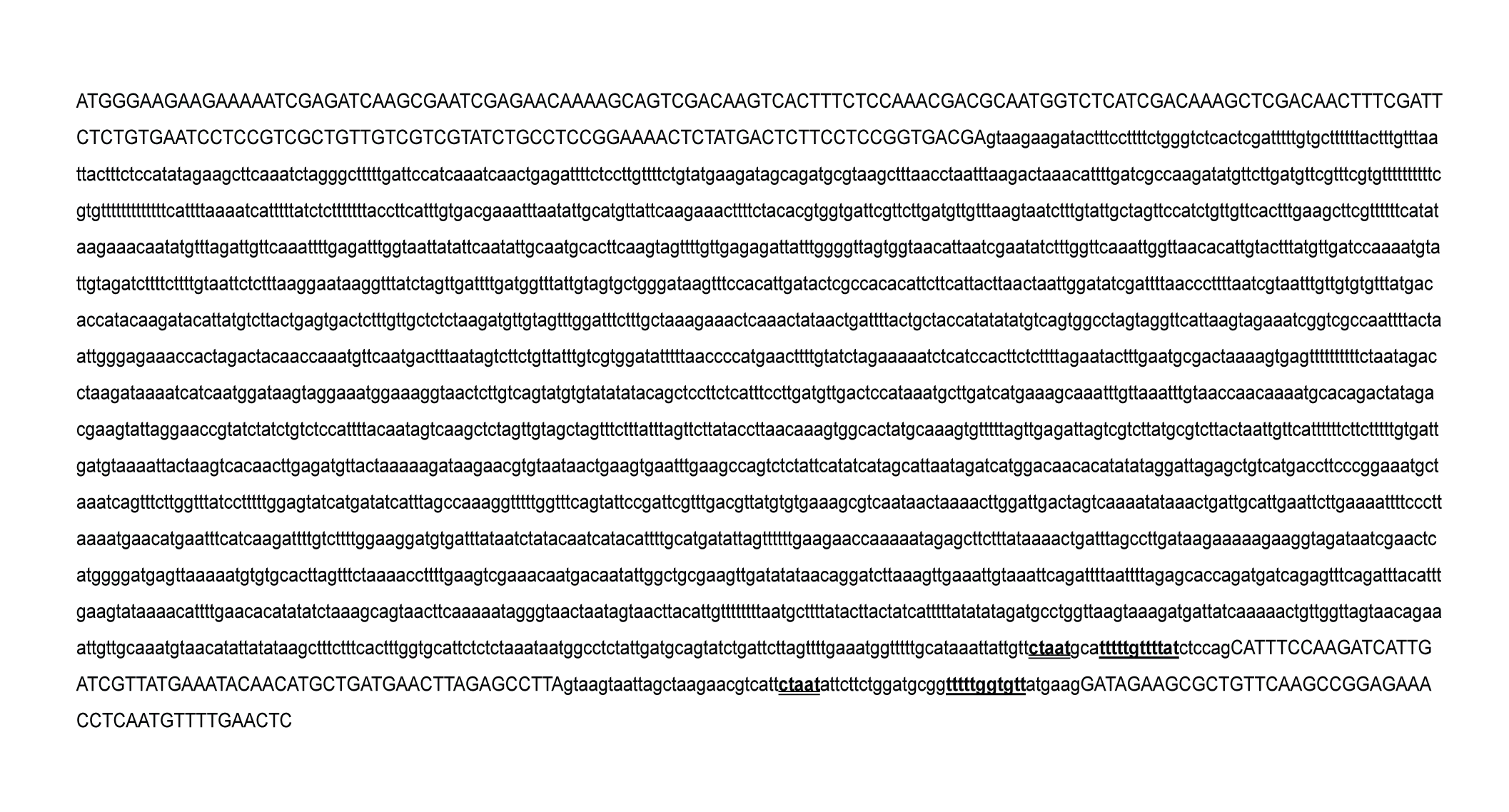

Supplement: Supplementary file 9 [file Image_8.TIF]

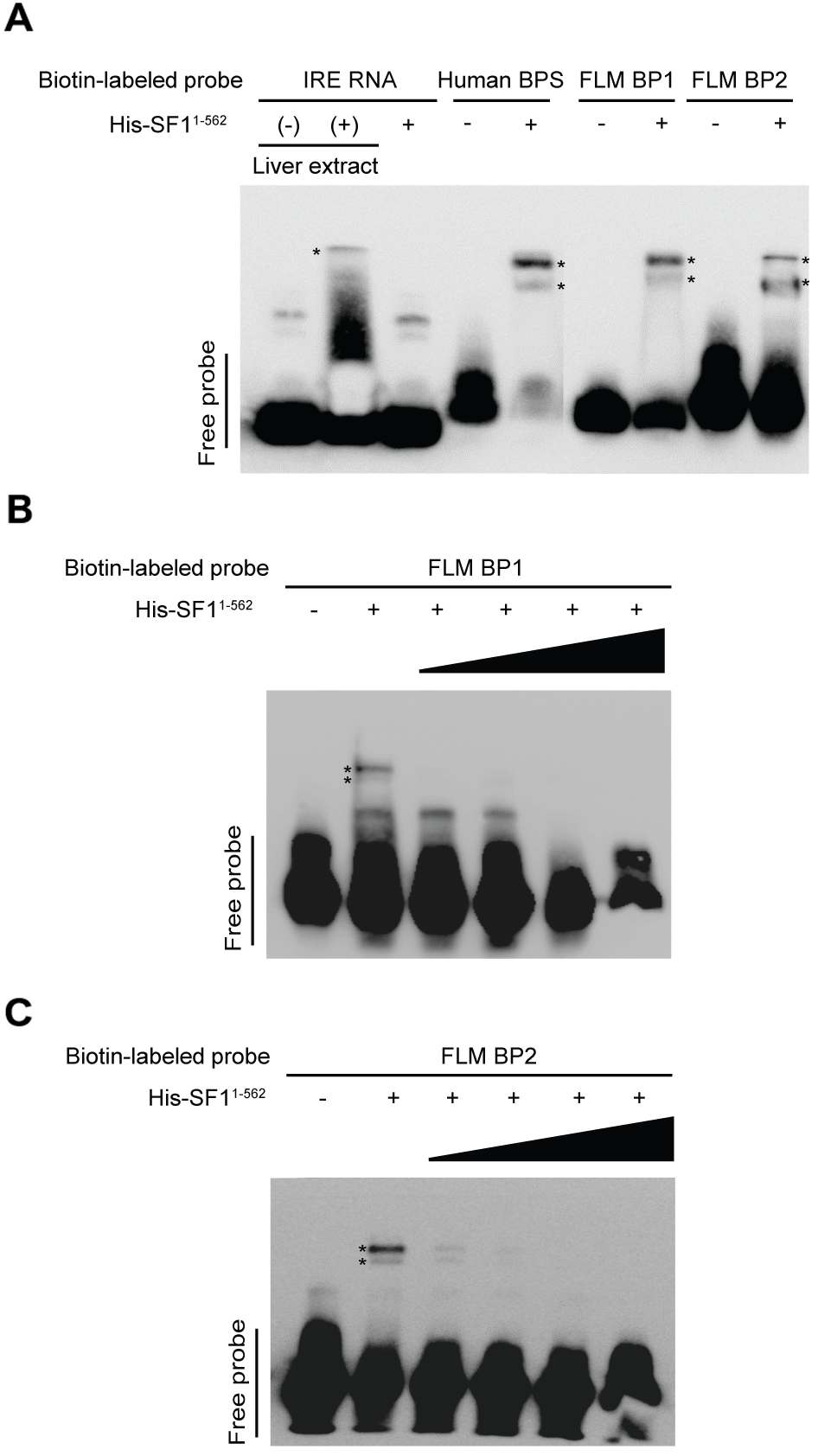

Supplement: Supplementary file 10 [file Image_9.TIF]

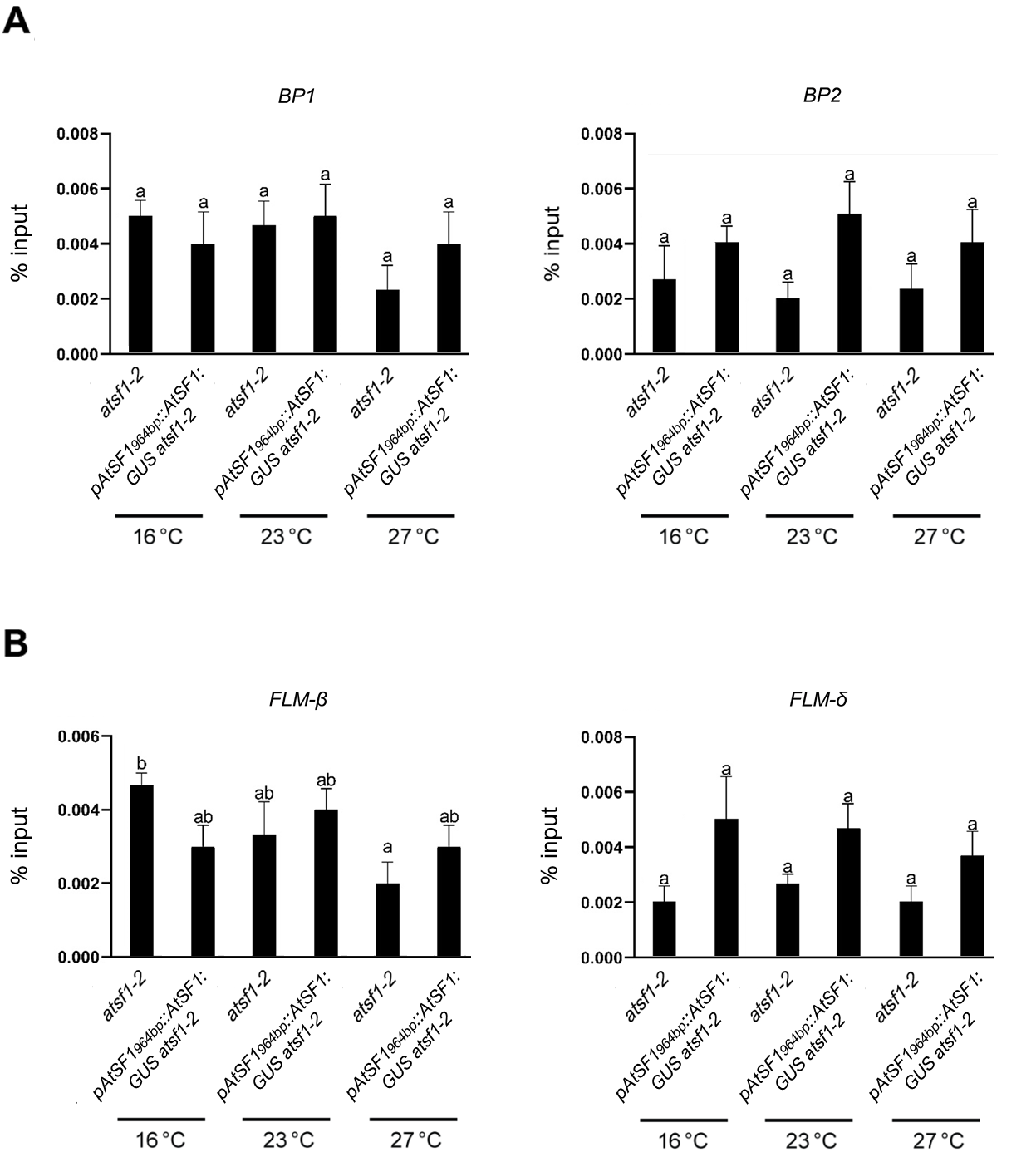

Supplement: Supplementary file 11 [file Image_10.TIF]

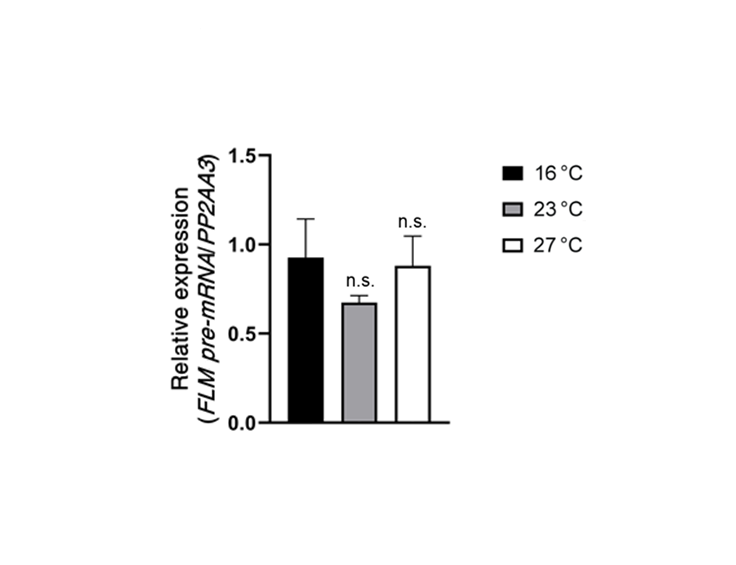

Supplement: Supplementary file 12 [file Image_11.TIF]
